# Runx2 drives Schwann cells repair phenotype switch through chromatin remodeling and Sox2 activation after nerve injury

**DOI:** 10.1186/s10020-025-01142-4

**Published:** 2025-03-21

**Authors:** Bo He, Shouwen Su, Zeyu Zhang, Zhongpei Lin, Qinglin Qiu, Yan Yang, Xiaoyue Wen, Zhaowei Zhu

**Affiliations:** 1 Orthopaedic Trauma and Joint Department, Department of Orthopedics, The Third Affiliated Hospital of Sun Yat-Sen University, Guangzhou, 510000 China; 2 Department of Dermatology, Guangzhou Dermatology Hospital, No. 56 Hengfu Road, Guangzhou, 510095 Guangdong China; 3 Department of Plastic Surgery, The First Affiliated Hospital of Sun Yat-Sen University, No. 58 Zhongshan Road 2, Guangzhou, 510080 China

**Keywords:** Pioneer transcription factor, Runx2, Schwann cell, Sox2, Epigenomic

## Abstract

**Background:**

The states of Schwann cells undergo significant shifts during nerve regeneration. Previous studies have shown the expression of Runx2 is locally upregulated within the affected areas. However, the regulatory mechanisms underlying its epigenetic control remain unclear.

**Methods:**

To investigate the epigenetic mechanisms through which Runx2 influences the phenotypic transition of repair Schwann cells. Runx2 siRNA fragments and Runx2 overexpression plasmids were constructed. Healthy adult Sprague–Dawley (SD) rats weighted 100–150 g, regardless of sex, were randomly selected. Following the establishment of a sciatic nerve crush injury model, samples were collected for qPCR analysis at 4 and 7 days post-injury. In vitro, the alterations in cell morphology, proliferation, apoptosis, and the ability to promote neural regeneration following the downregulation or upregulation of Runx2 in Schwann cells were assessed. A comprehensive analysis of transcriptome data, ATAC sequencing, and CUT&Tag sequencing of histones and transcription factors in SCs after Runx2 overexpression, along with single-cell RNA sequencing data from GSE216665 and Sox2 overexpression data from RSC96 in GSE94590, was conducted to elucidate the mechanism of action of Runx2, which was subsequently validated using dual luciferase assays.

**Results:**

Runx2 expression increased locally during the early stages of injury, primarily localized within Zhu Schwann cells (Zhu SCs). Runx2-overexpressing Schwann cells, when cultured in vitro, underwent a transformation from long, spindle-shaped He Schwann cells (He SCs) to flat, rounded Zhu SCs. Multi-omics analysis indicated that Runx2-OE may positively feedback-regulate its expression by opening transcriptional regulatory regions and binding to its own gene regulatory domains. Furthermore, it could also activate transcription factors such as Sox2, transitioning them from a transcriptionally silent to an active state, thereby enhancing Sox2 expression and synergistically regulating the phenotypic transition of Schwann cells.

**Conclusions:**

Runx2 can activate and recruit downstream stemness factors, such as Sox2, by modulating chromatin accessibility and histone modification status within Schwann cells, thereby promoting and maintaining the timely phenotypic transformation of Schwann cells following injury.

**Graphical Abstract:**

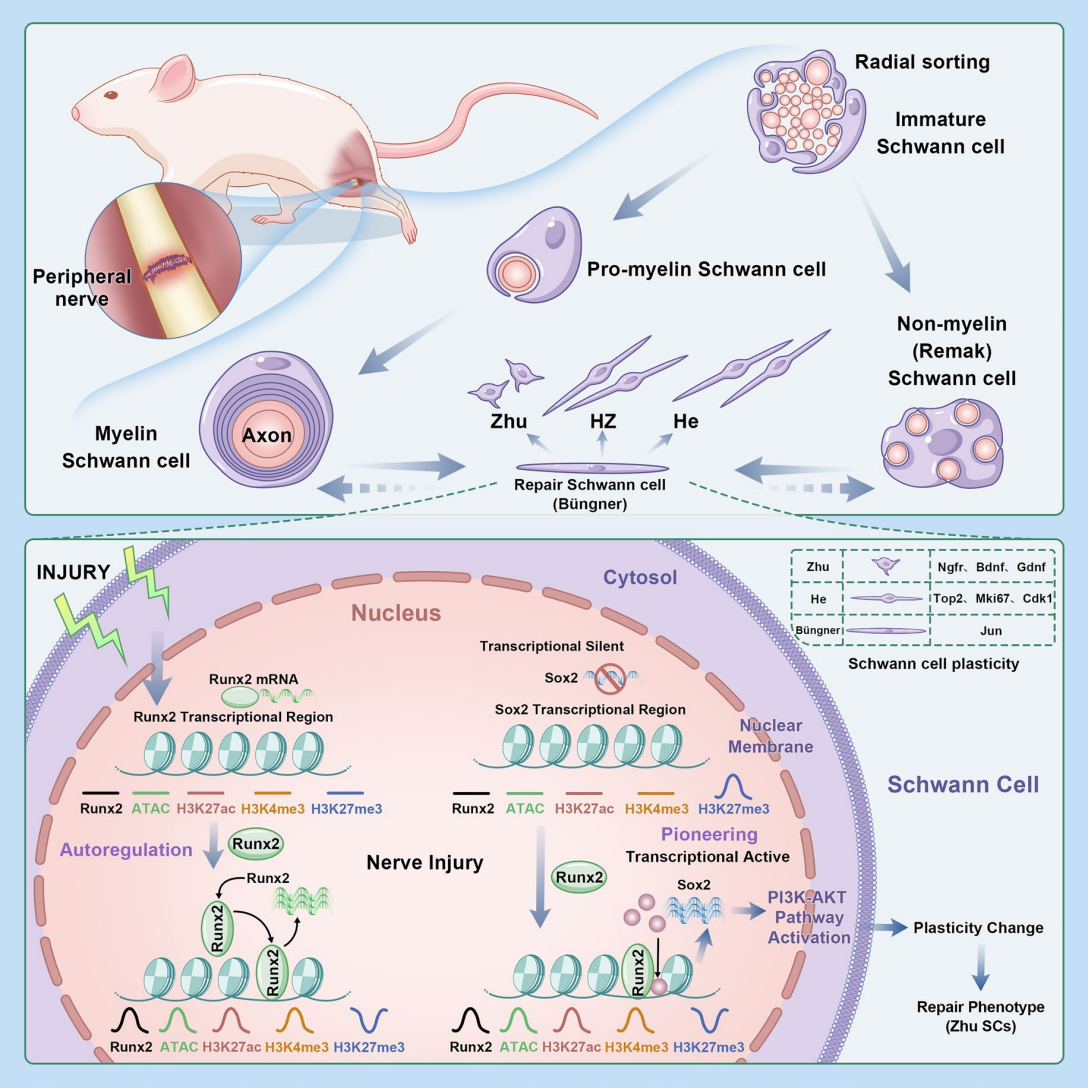

**Supplementary Information:**

The online version contains supplementary material available at 10.1186/s10020-025-01142-4.

## Highlights

- 1. Novel Autoregulatory Mechanism of Runx2: We uncovered that Runx2 establishes a rapid positive feedback loop, enabling swift self-activation and sustained high expression. This autoregulatory mechanism represents a crucial molecular switch in the injury response pathway.
- 2. Pioneer Factor Activity of Runx2 in Chromatin Remodeling: We elucidated the mechanistic role of Runx2 as a pioneer transcription factor, demonstrating its capacity to modulate chromatin accessibility, Orchestrate histone modifications and Facilitate the recruitment and binding of additional transcription factors.
- 3. Runx2-Mediated Activation of Sox2: We identified a novel regulatory axis wherein Runx2 activates the transcriptional silent stemness factor Sox2, and this Runx2-Sox2 cooperative interaction drives rapid phenotypic conversion of Schwann cells into repair phenotype post-injury.

**Supplementary Information:**

The online version contains supplementary material available at 10.1186/s10020-025-01142-4.

## Background

Peripheral nerve regeneration following injury remains a significant challenge in modern medicine. Schwann cells (SCs) play crucial roles in this regenerative process, with their functional state dynamically changing in response to nerve injury and repair (He et al. 2022). The state of SCs is regulated by various transcription factors (Li et al. 2021). Arthur-Farraj reported that activation of the transcription factor c-Jun in SCs is a global regulator of Wallerian degeneration because it governs major aspects of the injury response (Arthur-Farraj et al. 2012). Hung et al. conducted ChIP sequencing analysis on injured peripheral nerves in rats and discovered that upon activation, c-Jun binds to the enhancer sequence of *Runx2*, inducing its upregulation and participating in the regulation of SC activation (Hung et al. 2015). Similarly, Lovatt and coworkers, via single-cell sequencing, reported comparable results at 3, 12, and 60 days after sciatic nerve crush injury in rats (Lovatt et al. 2022), suggesting that SCs had differentiation states following peripheral nerve injury.

The Runt-related transcription factor (Runx) family comprises Runx1, Runx2, and Runx3, all of which possess a highly homologous Runt-related DNA binding domain. Runx2, also known as Osf2/Cbfa1, Pebp2αA, and AML-3, functions as a transcription factor and plays significant roles in tumorigenesis, migration, invasion, and bone and cartilage differentiation and maturation (Ozaki et al. 2018). Furthermore, Ding and coworkers demonstrated through in vitro experiments that Runx2 can activate the Akt/GSK3β pathway, promoting SCs differentiation and migration (Ding et al. 2018). These findings collectively underscore the importance of Runx2 in peripheral nerve regeneration and SCs regulation (Wang et al. 2022). In our previous studies, we performed next-generation sequencing (NGS) on the sciatic nerves of SD rats following crush injury. Subsequent validation through qPCR revealed significant upregulation of *Runx2* expression post-injury (He et al. 2021), suggesting its crucial role in the peripheral nervous system (Kalinski et al. 2020).

However, the current literature on the role of Runx2 in peripheral nerves, especially its mechanistic studies, is sparse. To delve deeper into the mechanisms by which *Runx2* affects SCs, *Runx2*-overexpressing (*Runx2*-OE) SCs were constructed in this study. Using assays for transposase-accessible chromatin (ATAC)-seq, histone modification (H3K27ac, H3K27me3) CUT&Tag, and transcription factor (Runx2, c-Jun, C/EBP-β, and CTCF) CUT&Tag with high-throughput sequencing of Runx2-OE SCs, along with subsequent validation, we aimed to elucidate the specific role and regulatory mechanisms of Runx2 in the dedifferentiation process of SCs. This study provides a comprehensive view of the epigenetic landscape and transcription factor dynamics involved in Runx2-mediated regulation within SCs.

## Methods

In this study, healthy adult Sprague‒Dawley (SD) rats weighing approximately 100–150 g, with no sex preference, were used to establish a model of nerve injury and to obtain dorsal root ganglia (DRGs). Additionally, neonatal SD rats aged 3–5 days, regardless of sex, were randomly selected for the extraction and culture of Schwann cells (SCs) from their sciatic nerves. The study received approval from the Experimental Animal Administration Committee of Sun Yat-Sen University. Measures were implemented to minimize the suffering of the animals during the experiments, ensuring that ethical standards in animal research were upheld, including appropriate anesthesia during surgical procedures, postoperative care to manage pain and discomfort, and humane endpoints to prevent unnecessary distress.

### Construction of the *Runx2* siRNA and *Runx2*-OE plasmid

*Runx2* siRNA and its control siRNA were constructed with the green fluorescent protein-encoding gene as the reporter sequence. The sequence of the gene target is 3′-GGT TCA ACG ATC TGA GAT T-5′. Moreover, an adenovirus carrying *the Runx2* sequence was constructed to overexpress *Runx2* (*Runx2*-OE), with the green fluorescent protein-encoding gene as a reporter sequence. As a control, the EGFP adenovirus was also used as a vector control. The specific sequences are shown in Figure S1.

### Establishment of a sciatic nerve injury model in SD rats and expression analysis of key genes

Adult SD rats of both sexes, weighing approximately 100‒150 g. The sciatic nerve crush model was established in both groups according to previous reports from the literature (Cai et al. 2021), and the nerves were harvested at 4-day post-injury (PI4d), and 7-day post-injury (PI7d). Normal sciatic nerves were harvested in both groups as normal control (NC). The sciatic nerve was clamped with a vascular clamp near the lower edge of the piriformis muscle for 30 s, followed by release. This clamping was repeated three times before closing the incision, thus to standardize injury severity. The repetition ensures methodological rigor, reproducibility, and irreversibility of damage for experimental validity. The injured parts were harvested at 4 days and 7 days after injury. These samples (n = 5) were processed and subjected to qPCR according to previous reports in the literature to assess changes in the expression of key injury-related genes, such as *Runx2*, *c-Jun*, *Egr2* and *Sox2* (Wu et al. 2023). The primer sequences are shown in Table S1, and the process is briefly described below. First, RNA was extracted with TRIzol, and RNA concentration was assessed with a NanoDrop system. The final concentration was adjusted to 200 ng/μL RNA (1 μg from each sample) with the RevertAid First Strand cDNA Synthesis Kit. An appropriate amount of cDNA was amplified via FastStart Universal SYBR Green Master Mix in a StepOnePlus real-time PCR instrument. The specific procedure is described in the system manual. Three replicates per sample were analysed. The expression levels of all target mRNAs were normalized to those of Gapdh. The specificity of the RT‒PCR results was confirmed via routine agarose gel electrophoresis and melting curve analysis. The 2^−ΔΔCt^ method was used to calculate relative gene expression levels.

### Single-cell sequencing analysis of SCs subtypes following injury

Single-cell RNA sequencing (scRNA-seq) data of rat sciatic nerves were obtained from the GEO database (http://www.ncbi.nlm.nih.gov/geo/, accession number GSE216665). The dataset includes samples from normal sciatic nerves (NCs) and crushed nerves at 3 days (PI3d), 12 days (PI12d), and 60 days (PI60d) post-injury. Following previously reported methods (Lovatt et al. 2022; Kalinski et al. 2020), SCs were annotated on the basis of the expression of the marker genes Sox10, Plp1, and S100b. SCs were further categorized into subtypes on the basis of gene expression patterns reported in the literature. The classification criteria are summarized in Table S4. SCs subtypes were analysed across different time points (NC, PI3d, PI12d, and PI60d) to elucidate the dynamic changes in SCs populations following nerve injury. Standard scRNA-seq analysis pipelines, including quality control, normalization, dimensionality reduction, and clustering, were employed and are briefly described below. The scRNA data were processed and analysed via the Seurat package (version 5.0) in R. Raw data from GSE216665 were downloaded and subjected to quality control to remove low-quality cells and potential doublets, excluding cells with unique feature counts greater than 6000 or less than 200 and cells with greater than 15% mitochondrial content. Normalization was performed via the “LogNormalize” method, followed by identification of the top 2000 highly variable genes. Principal component analysis (PCA) was used for dimensionality reduction, selecting the principal components on the basis of the elbow plot. Dataset integration was performed via Seurat's “Integration” workflow and canonical correlation analysis (CCA) to correct for batch effects. Cell clustering was achieved with the Louvain algorithm in the “FindClusters” function, and clusters were visualized via uniform manifold approximation and projection (UMAP). Next, an integrated method was chosen to annotate all the clusters, which combined three algorithms, namely, GPTCelltype (version 1.1.1), SingleR (version 1.0.0) and scCATCH (version 3.1.1). The reference cell marker genes were acquired and generalized from previous reports (Communications Biology 5.1 (2022): 1105). The annotated results were visualized via Seurat and ggplot2 (version 3.5.1). The subclusters of selected cell types were also annotated via the same strategy described above. Differential gene expression analysis was performed with the “wilcoxauc” function, which was supported by the R package RPresto (version 1.4.6). For each cell type, marker genes were defined as differentially expressed mRNAs (DEmRNAs) with an adjusted p value < 0.05 and a log2-fold change > 0.25 compared with the remaining cell types. For each group, DEmRNAs of each cell type were defined as genes with an adjusted p value < 0.05 and a log2-fold change > 0.25 between the two groups. Gene ontology, KEGG and GSEA enrichment analyses of DEmRNAs were performed via the R package clusterProfiler (version 4.6.0). To predict and map the differentiation of SCs, Monocle2 was used to perform pseudotime trajectory analysis and determine the translational relationships among the three cell types. The Monocle2 plot_pseudotime_heatmap function was used to assess the crucial roles of various genes in the differentiation process. Genes with a q value < 0.01 were considered significantly changed genes and were identified via the differential GeneTest function in Monocle2.

### In vitro observation of the changes in Runx2 expression after Runx2 overexpression or inhibition

In accordance with previous methods (Wong et al. 2023), SCs from 3 to 5-day-old neonatal SD rats were isolated and purified. P0 cells were cultured at 37 °C in a 5% CO_2_ incubator until they reached 90% confluence. According to the guidelines in the *Runx2* siRNA manual, 5 μL (20 μM) of siRNA transfection reagent was added to 250 μL of Opti-Men serum-free medium. Moreover, 5 μL of Lipofectamine 3000 Reagent was added to 250 μL of Opti-Men for 5 min. The two tubes of reagents were mixed and allowed to stand for 20 min. The serum-containing medium in the prepared 6-well plate was removed, replaced with transfection solution and added to the Opti-Men until a volume of 2 ml was reached. The plate was allowed to stand for 4 h, after which the medium was replaced with complete medium. A group of wild-type P0 SCs were established and incubated in an incubator for 72 h.

The purified P0 SCs were randomly divided into the *Runx2*-OE group and the vector (EGFP empty vector) group. SCs were transduced with *Runx2*-OE or vector adenovirus at a concentration of MOI = 200 for 72 h of incubation.

Changes in cell morphology were observed via laser confocal microscopy. Cell viability was determined via the EdU method, and cell apoptosis was observed via the TUNEL method. RNA was extracted from the cells of different groups, and the expression of *Runx2* was measured via qPCR. The primer sequences are shown in Table S1. With GAPDH as the housekeeping gene, the expression levels of related genes were calculated via the 2^−ΔΔCt^ method. The above experiment was repeated 3 times for each group. Data were collected for statistical analysis and plotting.

### Acquisition of dorsal root ganglia (DRGs) from rats and establishment of a coculture model

In accordance with previous reports in the literature (Qiu et al. 2015), neonatal SD rats 3–5 days old were randomly selected. The DRG tissues were obtained under a posture microscope. After the excess tissue was removed, the tissues were transferred to precooled D-Hank’s buffer and cocultured with the SCs in the aforementioned *Runx2*-OE and EGFP groups (n = 3). The cells were cocultured at 37 °C in 5% CO2 in an incubator for 72 h and then fixed with 4% PFA. Immunofluorescence staining was performed with NF. Nuclei were labelled with Hoechst 33,342 (Hoe). The staining was observed under a laser confocal microscope, and images were obtained for subsequent analysis.

### mRNA sequencing analysis of SCs overexpressing Runx2

High-throughput transcriptome sequencing was performed by Shanghai Biotechnology Corporation (Shanghai, China). In brief, SCs (n = 2) in the aforementioned *Runx2*-OE and EGFP groups were obtained. The RNA was extracted, and cDNA was obtained after amplification via the SMART-Seq^®^ HT Kit. After qualified electrophoresis with a Qubit and an Agilent Bioanalyzer 2100, the sequencing sample library was constructed. In accordance with the corresponding process shown in cBot and the Illumina User Guide, paired-end sequencing was conducted in PE150 sequencing mode. Real-time data analysis was performed. The reads were aligned to Rnor_6.0 via HISAT2 (version 2.0.4). During transcriptome sequencing, the reads were converted into fragments per kilobase of transcript per million mapped reads (FPKM) values to normalize the gene expression level (Liu et al. 2018). Analysis of differentially expressed genes (DEmRNAs) among samples was performed via the edgeR method. For downstream analyses, only genes with a |log_2_FoldChange(FC)|> 1 were included (*p* value < 0.05).

### ATAC sequencing analysis of SCs overexpressing *Runx2*

ATAC-Seq was performed according to the user guide of the Hieff NGS^®^ ATAC-Seq Library Prep Kit and by Guangzhou Huayin Health Medical Group Co., Ltd. (Guangdong, China). Briefly, the cells were harvested and counted at room temperature. Approximately 50,000 cells were pretreated with DNase for 30 min at 37 °C to remove free-floating DNA and to digest DNA from dead cells. Then, the cells were resuspended in a PCR tube and centrifuged for 5 min at 4 °C and 2300 RPM. The cell pellets were resuspended in 50 μL of cold lysis buffer and incubated on ice for 5 min to carry out the lysis reaction. The supernatant was removed, and 50 μL of transposition mixture was added directly to the pellet. Transposition reactions were executed at 37 °C for 30 min in a PCR amplifier. After the PCR, the PCR tube was centrifuged immediately. A total of 5 μL of termination mixture was added to the PCR tube, which was incubated at room temperature for 5 min. Afterwards, 130 μL of extracted DNA beads were added to a 55 μL PCR tube for size selection. The supernatant was transferred to a new tube, and 10 µL of fresh beads was added to capture fragments. The fragment distribution was determined by using a Bioptic Qsep400 Analyzer. DNA fragments ranging from 250 to 350 bp were retained for library preparation. The library products were sequenced via the Illumina NovaSeq 6000 system. Three biological replicates were sequenced for the Runx2-OE group and EGFP vector group.

ATAC-Seq raw reads were filtered and trimmed by using Trimmomatic (version 0.39) to remove the sequencing adaptors and low-quality reads. The quality control data were processed by using multiQC (version 1.22.1). After quality control, the clean reads were mapped to the reference genome UCSC Rn6 by using Bowtie2 (version 2.5.1). The R package ATACseqQC (version 1.28.0) was chosen to perform postalignment quality assessment. Only the uniquely mapped reads were retained for the next step of the analysis. The bam file generated from the unique mapped reads was sorted via samtools (version 1.20), and the sorted bam file was chosen for peak calling by using MACS2 (version 2.2.9.1) with default parameters. The R package Diffbind (version 3.14.0) was chosen to perform the differential peak analysis. Peaks were considered differently accessible if the p value was < 0.05. Differential peaks were annotated via the annotatePeaks.pl tool from HOMER software (version 4.11). Motif enrichment analysis of differential peaks was performed with the findMotifsGenome.pl tool from HOMER software (version 4.11). Functional enrichment was performed on genes associated with differential peaks via the R package clusterProfiler (version 4.6.0). The sorted bam file was converted into bigwig format by using the bamCoverage tools of deepTools (version 2.0). The metagene plot was generated via computeMatrix tools and plotted via plotProfile tools, both of which were from deepTools (version 2.0). A Venn diagram was created to determine the intersection between DEmRNAs in the ATAC-seq analysis and those in the mRNA-seq analysis. GSEA was used to determine hallmark gene sets and cell type signature gene sets.

### Histone and transcription factor CUT&Tag library construction

The CUT&Tag assay was performed as described previously with modifications (Kaya-Okur et al. 2019) and was performed by Guangzhou Huayin Health Medical Group Co., Ltd. (Guangdong, China). Briefly, 1 × 10^6^ cells were washed twice gently with cell wash buffer. A total of 10 μL of Concanavalin A-coated magnetic beads were added to each sample, and the mixture was incubated at room temperature for 10 min. The unbound supernatant was removed, and the bead-bound cells were resuspended in Dig wash buffer and incubated with a 1:50 dilution of primary antibody (ATAC, H3K27ac, H3K4me3, H3K27me3, Runx2, c-JUN, C/EBP-β, or CTCF) on a rotating platform overnight at 4 °C. The primary antibody was removed using a magnet stand. The secondary antibody was diluted 1:100 in Dig wash buffer, and the cells were incubated at room temperature for 60 min. The cells were washed with a magnet stand 2–3 times in Dig wash buffer. A 1:100 dilution of the pA-Tn5 adapter complex was prepared in Dig-med buffer and incubated with the cells at room temperature for 1 h. The cells were subsequently washed 2–3 times for 5 min in 1 mL of Dig-med buffer. Then, the cells were resuspended in tagmentation buffer and incubated at 37 °C for 1 h. DNA was purified via phenol‒chloroform-isoamyl alcohol extraction and ethanol precipitation. DNA fragments ranging from 250 to 350 bp were retained for library preparation. The library products were sequenced by using the Illumina NovaSeq 6000 system.

CUT&Tag raw reads were filtered and trimmed by using Trimmomatic (version 0.39) to remove the sequencing adaptors and low-quality reads. The quality control data were processed via multiQC (version 1.22.1). After quality control, the clean reads were mapped to the reference genome UCSC Rn6 by using Bowtie2 (version 2.5.1). The R package ATACseqQC (version 1.28.0) was chosen to perform postalignment quality assessment. Only the uniquely mapped reads were retained for the next step of the analysis. The bam file generated from the unique mapped reads was sorted by using samtools (version 1.20), and the sorted bam file was chosen for peak calling by using MACS2 (version 2.2.9.1) with default parameters. The R package Diffbind (version 3.14. 0) was chosen to perform the differential peak analysis. Peaks were called differently if the p value was < 0.05. Differential peaks were annotated via the annotatePeaks.pl tool from HOMER software (version 4.11). Motif enrichment analysis of differential peaks was performed by using the findMotifsGenome.pl tool from HOMER software (version 4.11). Functional enrichment analysis was performed on the genes associated with differential peaks via the R package clusterProfiler (version 4.6.0). The sorted bam file was converted into bigwig format by using the bamCoverage tools of deepTools (version 2.0). The metagene plot was generated via computeMatrix tools and plotted via plotProfile tools, both of which were from deepTools (version 2.0). The overlap peaks of ATAC-Seq and the CUT&Tag assay were extracted by using bedtools intersect tools (version 2.28). IGV was used to visualize the peaks and signals of CUT&Tag targets.

In accordance with previous methods of the research group (He et al. 2021), Gene Ontology (GO) and Kyoto Encyclopedia of Genes and Genomes (KEGG) pathway enrichment analyses were performed. The significance level of each enriched GO term was calculated with Fisher’s test, and the significantly enriched terms were screened out with the test standard α set to 0.05. A heatmap of pathway terms with high DEmRNA enrichment was drawn with R language.

### Dual-luciferase assay to verify the binding sites of the *Runx2* peak region

To elucidate the specific mechanism by which *Runx2* acts as a pTF to regulate itself, we employed the FIMO component of the MEME software suite to scan and identify Runx2 binding sites within the *Runx2* peak region. On the basis of the identified binding site sequences, we designed the following luciferase reporter constructs, sequences of which were shown in Table S2-S3.

*Runx2* peak region verification:

Empty vector control: pGL3-Promoter;

E1/E1 Wild-Type vector: pGL3-Promoter/E1 (chr9 (18640850-18643595), 2745 bp);

E2 vector: pGL3-Promoter/E2 (chr9 (18708801-18709450), 650 bp);

E3 vector: pGL3-Promoter/E3 (chr9 (18755351-18756300), 950 bp);

E4 vector: pGL3-Promoter/E4 (chr9 (18769245-18771550), 2305 bp);

E1 mutant 1 vector: pGL3-Promoter/E1-Mut1;

E1 mutant 2 vector: pGL3-Promoter/E1-Mut2;

E1 mutant 3 vector: pGL3-Promoter/E1-Mut3;

These reporter constructs were prepared as viral vectors and cotransfected into 293 T cells along with either the *Runx2* overexpression plasmid (*Runx2*-OE) or a control plasmid (pcDNA3.1). The cells were cultured for 48 h posttransfection before fluorescence intensity was measured and data were collected. The luciferase reporter system was constructed by Dongzebio Co., Ltd. All results are presented as the mean ± standard error of the mean (SEM), with n = 4 plates of cells per condition.

### Microarray data analysis of Sox2-overexpressing (Sox2-OE) Schwann cell

The transcriptomic data of Sox2-OE and normal rat Schwann cells (RSC96) were retrieved from the GEO database (accession number: GSE94590) (Torres-Mejia et al. 2020). Raw data preprocessing and annotation were performed using R packages including tinyarray, GEOquery (version 2.74.0), and Annoprobe (version 0.1.7). A total of 3 Sox2-OE samples and 3 normal samples were subjected to subsequent analysis. Following gene quantification and filtering, differential gene expression analysis was conducted using the R package limma (version 3.62.1). Genes with |log2foldchange|> 1 and P-value < 0.05 were identified as significantly differentially expressed genes (DEGs). Based on the log2foldchange values, these DEGs were categorized into significantly upregulated and downregulated genes. Functional enrichment analysis was performed on both upregulated and downregulated genes using R packages clusterProfiler (version 4.14.4) and ReactomePA (version 1.50.0), followed by visualization. GO terms, KEGG and Reactome pathways with P-value < 0.05 were considered significantly enriched terms or pathways.

### Images, data processing and statistical analysis

Photoshop was used for image processing. ImageJ software was used for image data acquisition. SPSS and GraphPad Prism were used for all the statistical analyses. If the dataset fit a normal distribution and had a constant variance, one-way analysis of variance (ANOVA) combined with the Tukey multiple comparison method was performed to determine the specific differences among groups. The percentage data were analysed via the chi-square test with the test standard α set to 0.05.

The results of the luciferase reporter system were analysed via the Shapiro‒Wilk test, which indicated that the data satisfied the assumption of normality (P > 0.05). The Brown-Forsythe test suggested that the assumption of homogeneity of variances was not met (P > 0.05). Therefore, corrected one-way ANOVA (Brown-Forsythe and Welch ANOVA tests) was performed, followed by Dunnett’s test for pairwise comparisons; the WT or Vector group was used as the control and compared to the mutant groups.

## Results

### During the early stages of peripheral nerve injury, Runx2 expression is elevated in SCs at the site of injury

**Fig. 1:**
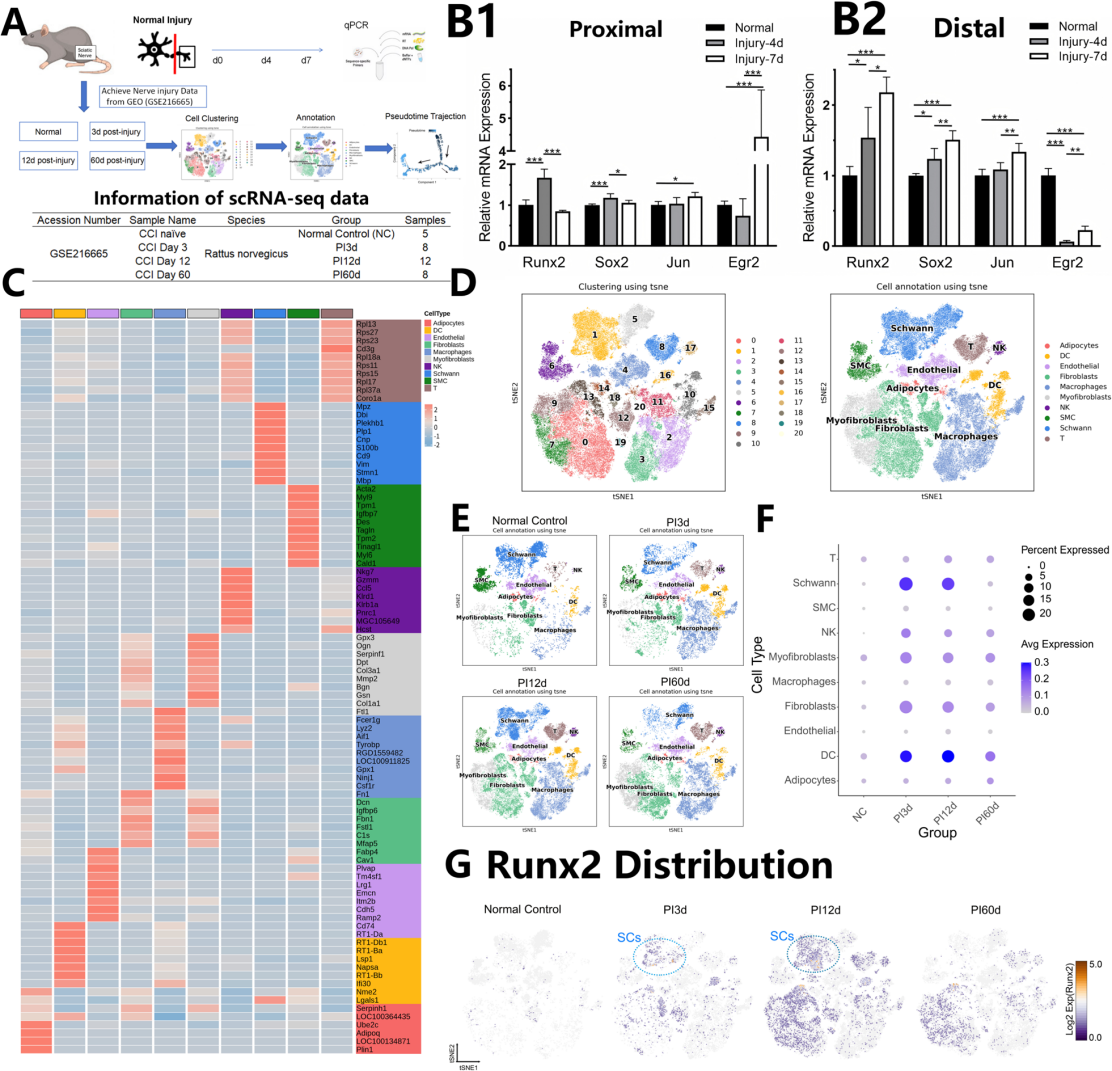
Molecular and Cellular Analysis of Sciatic Nerve Injury Response with Focus on Runx2 Expression. Data Source: GSE216665. **A** In vivo experimental design schematic and information of Single-cell RNA sequencing (scRNA-seq) data. **B** Injury-related mRNA Expression Analysis (n = 5). B1) Proximal stump temporal expression profile. B2) Distal stump temporal expression profile. **C** Heatmap visualization of cluster-specific marker genes in scRNA-seq data. **D** t-SNE plot showing spatial distribution of cell clusters. **E** t-SNE visualization of cell cluster distribution across injury time points. **F** Bubble plot showing cell type-specific Runx2 expression. Purple and white indicate gradually decreasing gene expression compared with the mean. The bubble size represents the proportion of cells expressing the gene within each cell type, with larger areas indicating higher expression proportions. **G** t-SNE visualization of Runx2 expression dynamics. Orange, white and purple indicate gradually decreasing gene expression compared with the mean. * indicates p < 0.05, ** indicates p < 0.01,*** indicates p < 0.005

After qPCR analysis of the proximal and distal ends of the injured nerve, it revealed that *Runx2* expression at the proximal end was significantly greater at 4 days postinjury than at preinjury (p < 0.01) but was lower at 7 days than at 4 days (p < 0.01) (Fig. 1B1). However, at the distal end, *Runx2* expression continuously increased, maintaining high levels up to 7 days postinjury (p < 0.01, Fig. 1B2).Compared with that of other SCs activation/dedifferentiation-related genes, the expression trend of *Runx2* was similar to that of the activation genes *Sox2* and *Jun* but opposite that of the differentiation-related gene *Egr2*, suggesting that *Runx2* might be involved in the differentiation of SCs.

In order to observe the cellular changes at the site of nerve injury, we reannotated the cells from GSE216665 (Lovatt et al. 2022). After quality control of the data, the data could be divided into 21 cell clusters (see Fig. 1D), and further classified them into adipocytes, dendritic cells (DC), endothelial cells, fibroblasts, macrophages, myofibroblasts, NK cells, smooth muscle cells (SMC), Schwann cells (SCs), and T-cells based on the molecular markers mentioned in the article (see Fig. 1C). The distribution of these cell populations at different injury times is shown in Fig. 1E.Compare the distribution changes of Runx2 in these cell populations, we found that Runx2 increased significantly from 3 days (PI3d) to 12 days (PI12d) after injury, and decreased at 60 days, but still remained higher than normal control (NC). The cell population with high expression of *Runx2* inlcuded DCs, Schwann cells, fibroblasts, etc. (see Fig. 1F). By visualizing expression of Runx2 through tSNE graph, it can also be found that *Runx2* was significantly increased in the SCs of PI3d and PI12d (see Fig. 1G).

### Runx2 can modulate the sequential dedifferentiation of myelin SCs into Zhu SCs and He SCs

**Fig. 2:**
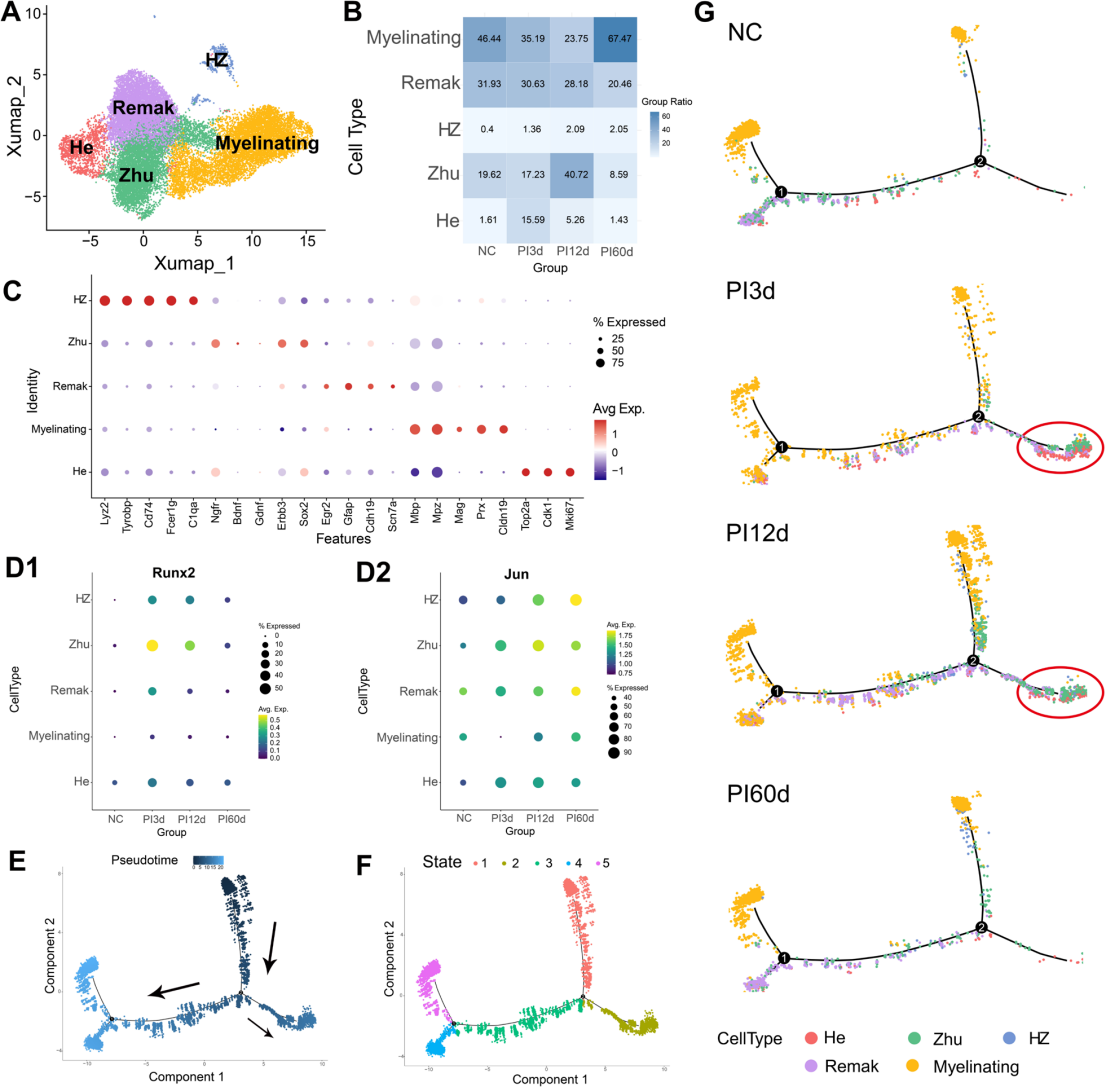
Single-cell RNA Sequencing (scRNA-seq) Analysis of Schwann Cell (SCs) Dynamics During Peripheral Nerve Injury and Regeneration. Data source: GSE216665.** A** Uniform Manifold Approximation and Projection (UMAP) visualization of distribution of distinct SCs subtypes post-nerve injury.** B** Temporal dynamics of SCs subtype composition. Stacked bar representation of subtype proportions across time points. Color intensity correlates with cell proportion. **C** Key gene expression profiles across SCs subtypes. Red indicates gene expression higher than average, and purple indicates gene expression lower than average. The size of the circle represents the proportion of cells expressing the gene within each subtype, with larger areas indicating higher expression proportions. **D** Differentiation-related gene (Runx2 and Jun) expression patterns. Yellow, green, blue, and purple indicate gradually decreasing gene expression compared with the mean. The bubble size represents the proportion of cells expressing the gene within each subtype, with larger areas indicating higher expression proportions. **E** Pseudotime trajectory analysis. Directional arrows indicating SCs developmental progression. Temporal evolution of cellular states. **F** Different states of SCs in the pseudotime analysis, with different colours representing distinct cellular states. **G** Pseudotime trajectory changes in different SCs subtypes at various time points post-injury. Red circle represented apprearance of newly transformed clusters after nerve injury. * indicates p < 0.05, ** indicates p < 0.01, *** indicates p < 0.005

We further extracted SCs and subdivided them into He SCs (corresponding to Kalinski’s SC1 and Lovatt's dividing SCs), Zhu SCs (corresponding to Kalinski’s SC3 and Lovatt's repairing SCs), myelinating SCs, and Remak SCs. Myelinating SCs in Fig. 2 include both actively myelinating and myelinated SCs, characterized by high expression of myelin proteins MBP and MPZ, distinguishing them from Kalinski's pro-myelinating SC3 population. Schwann cells that could not be categorized into these four types were classified as HZ SCs, potentially similar to Kalinski's SC2 or a subset of Lovatt's Transition SCs. The UMAP distribution of these cell types in the database is illustrated in Fig. 2A. Comparative analysis of highly expressed genes in each SCs subtype revealed that He SCs predominantly express proliferation-related genes such as *Mki67*. Zhu SCs primarily express genes associated with promyelination states and neurotrophic factors, including *Ngfr, Gdnf, Bdnf, Erbb3*, and *Sox2*. Myelinating SCs highly express myelin protein-related genes, notably *Mbp, Mpz, Prx*, and *Mag*. Remak SCs show elevated expression of differentiation-related genes, including *Sox2* and *Egr2* (Fig. 2C). He SCs, Zhu SCs, and HZ SCs emerged post-injury, with their cellular proportions fluctuating over time (Fig. 2B). In normal nerves, the predominant cell types were myelinating SCs (46.44%), Remak SCs (31.93%), and Zhu SCs (19.62%). At 3 days post-injury (PI3d), we observed a decrease in myelinating SCs (35.19%) and an increase in He SCs (from 1.61% to 15.59%), whereas the proportion of Zhu SCs remained relatively stable (17.23%). By 12 days after injury (PI12d), the proportion of myelinating SCs further decreased (23.75%), the proportion of He SCs decreased (5.26%), and the proportion of Zhu SCs markedly increased (40.72%). At 60 days after injury (PI60d), myelinating SCs had recovered to levels comparable to those of normal nerves (67.47% vs. 46.44%), whereas Remak SCs and Zhu SCs had lower proportions under these conditions than under normal conditions (20.46% vs. 31.93% and 8.59% vs. 19.62%, respectively).

Changes in key differentiation-related genes across various SCs subtypes at different time points post-injury are shown in Fig. 2D1-D2. *Runx2* was expressed primarily in Zhu, He, and HZ cells at PI3d and PI12d, with the highest expression noted in Zhu SCs at PI3d. As the injury time progressed, the expression of Runx2 in Zhu SCs gradually decreased, aligning with the expression levels in other SCs types at PI60d (Fig. 2D1). At PI3d, *Jun* was highly expressed in Zhu, He, and Remak cells, whereas its expression was significantly reduced in myelinating SCs. By PI12d, the expression of *Jun* in all SCs types continued to increase, and by PI60d, Jun was predominantly and persistently highly expressed in HZ and Remak cells (Fig. 2D2).

Pseudotime analysis of SCs data was conducted to assess the inferred temporal order of cell evolution (Fig. 2E) and perform pseudotime cell clustering (Fig. 2F). The analysis explored the differentiation and evolution of cells at different time points post-injury. The nerve in the control group (NC) predominantly contained myelinating SCs (yellow) and Remak SCs (purple). Within 12 days postinjury, the number of myelinating SCs gradually decreased, and the SCs transitioned into other SCs subtypes. Zhu SCs (green) appeared earliest, with a significant increase by PI12d, whereas He SCs (red) appeared later than Zhu SCs did, peaking in number at PI3d and subsequently decreasing. By PI60d, the pseudotime trajectory resembled that of normal nerves (Fig. 2G).

### Overexpressing Runx2 in SCs resulted in cell plasticity change and suppressed proliferation in vitro

**Fig. 3:**
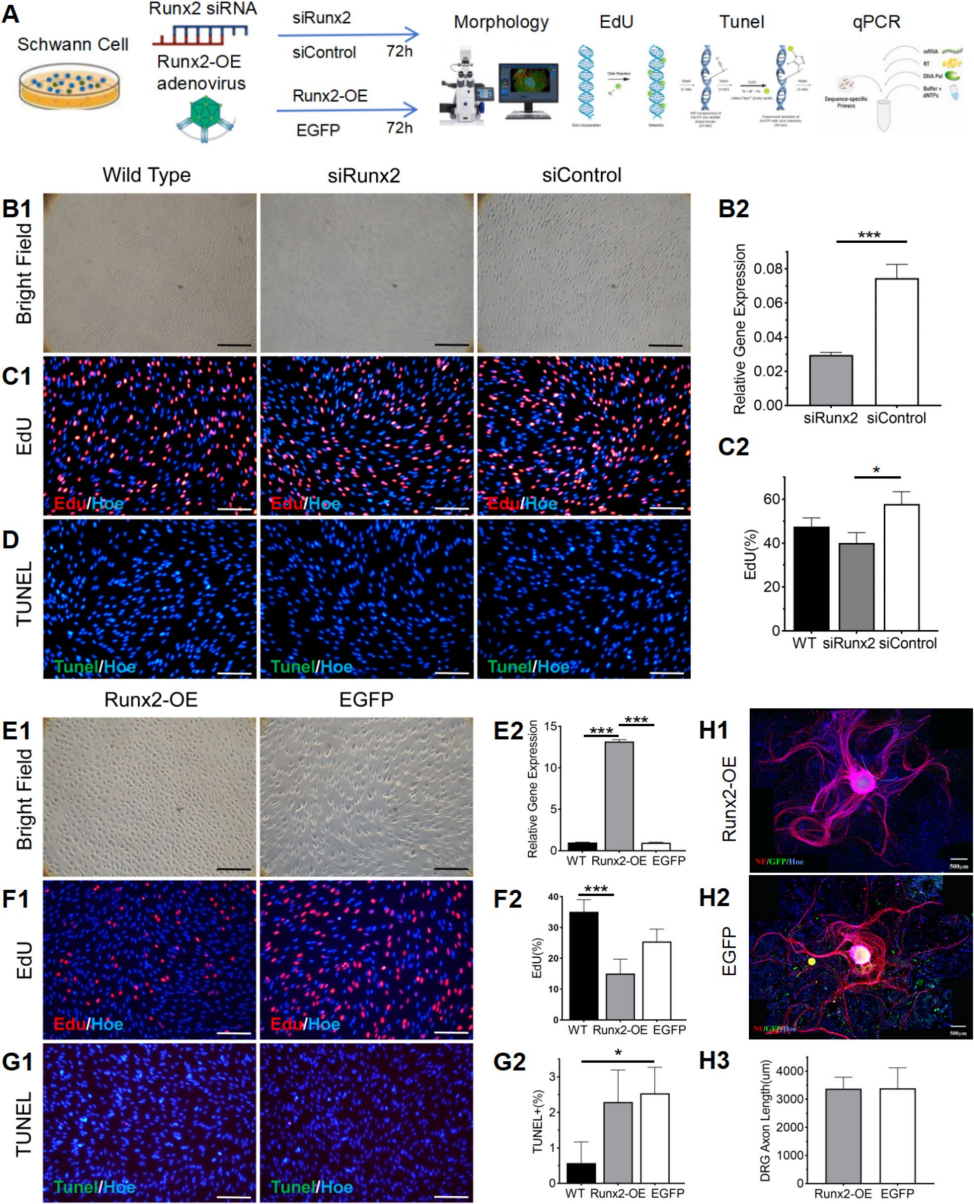
Functional Impact of Runx2 Modulation on Schwann Cell (SCs) Biology and Axonal Growth in SCs-DRG Coculture System. **A** Experimental design schematic for in vitro studies (n = 3). **B** Runx2 interference Analysis. **B1** Phase-contrast microscopy of SCs morphology. **B2** Quantitative PCR validation of Runx2 expression post-siRNA transfection. **C** Cell Proliferation Assessment (Runx2 interference). **C1** EdU incorporation assay (red: proliferating cells). **C2** Quantification of proliferation rates. **D** Apoptosis Analysis via TUNEL assay visualization (Runx2 interference, green: apoptotic cells). **E** Runx2-OE Analysis. **E1** Bright-field microscopy of SCs morphology post-transfection. **E2** Quantitative PCR confirmation of Runx2-OE. **F** Cell Proliferation Assessment (Runx2-OE). **F1** EdU incorporation assay (red: proliferating cells). **F2** Quantification of proliferation rates. **G** Apoptosis Analysis (Runx2-OE). **G1** TUNEL assay visualization (green: apoptotic cells). **G2** Quantification of apoptotic rates. **H** SCs-DRG Coculture Analysis. **H1** Fluorescence imaging of Runx2-OE SC cocultures. **H2** Fluorescence imaging of EGFP SCs cocultures. **H3** Quantitative comparison of axonal extension. In the above images, WT represents the wild type, siRunx2 represents the *Runx2* inhibition group, siControl represents the inhibition control group, *Runx2*-OE represents the *Runx2* overexpression group, and EGFP represents the empty vector group. In all fluorescence images, blue indicates Hoechst (Hoe)-stained nuclei. Red in H1 and H2 represents NF + axons, whereas green represents transfected SCs. The scale bars in B1, C1, D, E1, F1, and G1 represent 100 μm; the scale bars in H1 and H2 represent 500 μm. * indicates p < 0.05, ** indicates p < 0.01, *** indicates p < 0.005

At 72 h posttransfection, the expression of *Runx2* was significantly lower in cells treated with siRunx2 than in those in those treated with siControl (p < 0.01, Fig. 3B2). Optical microscopy revealed that WT primary SCs had smaller cell bodies with slender protrusions on both sides, forming a network-like structure with adjacent cells. After *Runx2* siRNA was applied, no significant changes in cell morphology were observed (Fig. 3B1). The results of the EdU assay revealed changes in SCs proliferation capacity in the Runx2 siRNA group compared with the control group (p < 0.05, Fig. 3C1-C2). TUNEL assays revealed no significant changes in the number of apoptotic SCs compared with that in the control group (Fig. 3D).

After primary SCs were infected with Runx2-OE and EGFP adenoviruses, the expression of *Runx2* in the *Runx2*-OE group was significantly upregulated compared with that in the EGFP group (p < 0.05, Fig. 3E2). Under the microscope, SCs in the *Runx2*-OE group appeared larger and flatter than those in the EGFP group, with retracted protrusions at both ends and the disappearance of the network-like structure between cells (Fig. 3E1). EdU staining revealed a significant reduction in cell viability in the *Runx2*-OE group compared with the EGFP group (p < 0.05, Fig. 3F1-F2). TUNEL experiments revealed that the number of apoptotic SCs increased in the viral transduction group compared with that in the WT group, although there was no significant difference between the *Runx2*-OE and EGFP groups (p > 0.05, Fig. 3G1-G2).

After *Runx2*-OE SCs were cocultured with Dorsal root ganglions (DRGs) for 72 h, no significant difference in axonal growth length was detected in this group compared with the EGFP group (p > 0.05, Fig. 3H1-H3).

### Runx2 influences Schwann cell plasticity by reducing expression of genes related to SCs differentiation

**Fig. 4:**
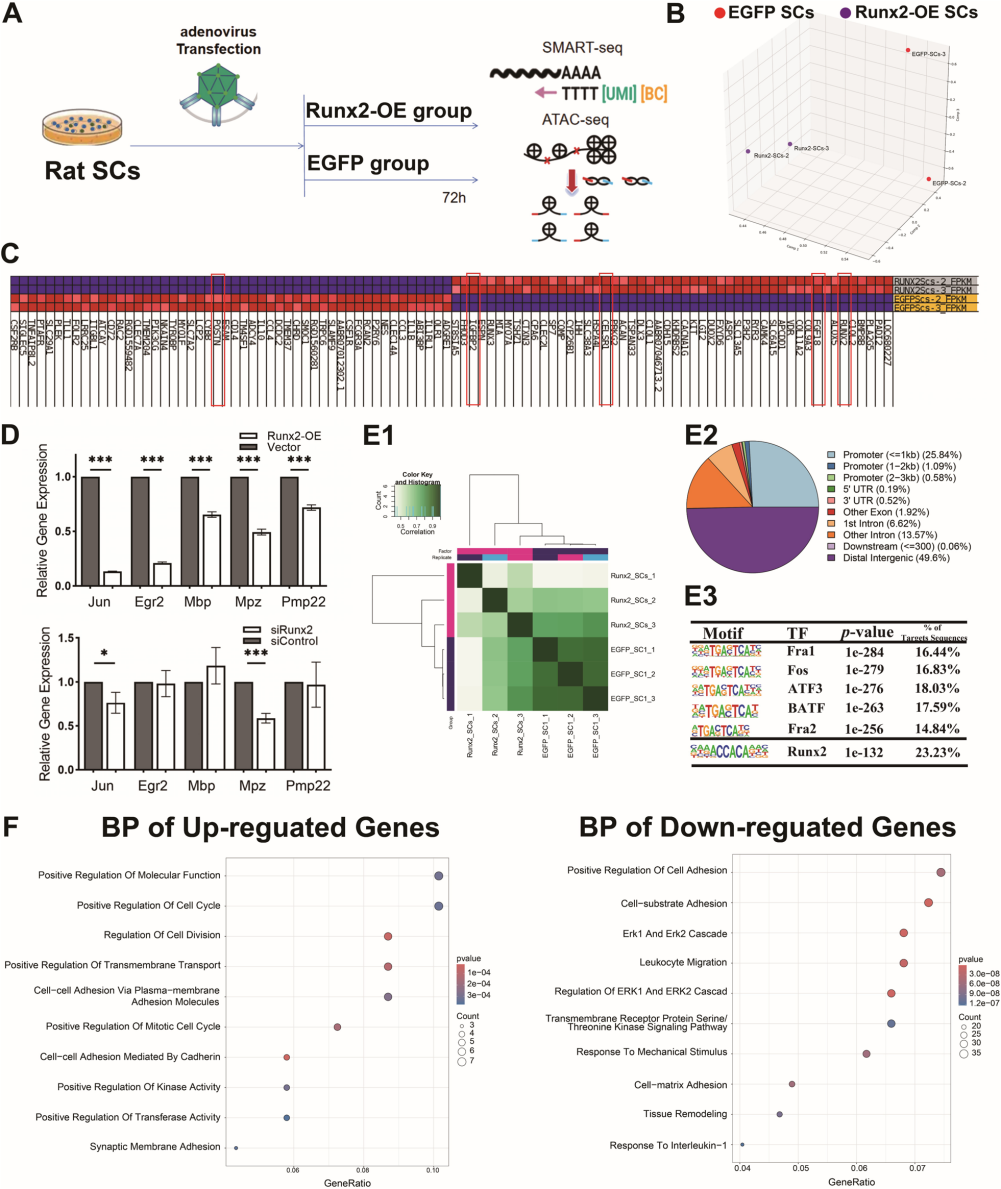
Integrated Analysis of Transcriptome and Chromatin Accessibility in Runx2-Overexpressing Schwann Cells (Runx2-OE SCs). **A** Schematic representation of experimental design and workflow. **B** Three-dimensional Principal Component Analysis (PCA) visualization of transcriptome profiles (RNA-seq, n = 2) comparing Runx2-OE and EGFP control groups. **C** Hierarchical clustering heatmap of differentially expressed mRNAs (DEmRNAs). Red color indicates upregulation in Runx2-OE, and blue indicates downregulation. The red boxes highlight key mRNAs related to nerve regeneration. **D** Quantitative PCR validation of differentiation and myelin-related markers after Runx2-OE or inhibition (n = 3). **E** ATAC-seq Analysis of Runx2-OE SCs (n = 3). **E1** PCA plot demonstrating chromatin accessibility patterns across experimental groups. **E2** Pie chart showing genomic distribution of differential ATAC-seq peaks. **E3** HOMER motif analysis of enriched transcription factor binding motifs within differential peaks (p < 0.05). **F** Integrated GO enrichment analysis (Biological Process, BP) of concordantly regulated genes in ATAC-seq and RNA-seq data. The circle size represents the number of genes, and the colour gradient from red to blue indicates increasing p values. * indicates p < 0.05, ** indicates p < 0.01,*** indicates p < 0.005

Smart-seq mRNA sequencing was performed on SCs from the *Runx2*-OE and EGFP groups. The results revealed that 2662 genes (DEmRNAs) were differentially expressed in the *Runx2*-OE SCs; the 1317 upregulated genes included *Runx2, FGF18, Ceslr1,* and *IGFBP2*, and the 1345 downregulated genes included *POSTN and PIK3G* etc. (Figs. 4B, C). qPCR was used to measure the expression of genes related to differentiation (*Jun, Egr2*) and myelination (*Mbp, Mpz, Pmp22*). The results indicated a significant downregulation of *Jun, Egr2, Mbp, Mpz*, and *Pmp22* in *Runx2*-OE SCs (p < 0.05). In siRunx2 SCs, the expression of *Mpz* was significantly downregulated, whereas the expression of other genes was greater than that in the Runx2-OE group but not significantly different from that in the siControl group, suggesting that *Runx2* may interferes with the myelination process via *Mbp* (Fig. 4D).

ATAC-seq was also performed in the *Runx2*-OE and EGFP groups. In *Runx2*-OE SCs, 5396 regions presented peak expression increases after *Runx2* upregulation, whereas 765 regions presented weakened or even absent peaks posttransduction. The majority of peak regions were concentrated in distal intergenic regions (49.6%), followed by short promoter regions (≤ 1 kb, 25.84%) and other intronic sequences (13.57%). HOMER was used to predict motifs in peak regions and revealed that TFs such as Runx2, Fra1, Fos, ATF, BATF, and Fra2 could bind to peaks, with Runx2 binding up to 23.23% of the sites in the open peak regions (Fig. 4E), suggesting that downstream gene expression is closely related to the direct binding of Runx2.

The ATAC-seq and mRNA sequencing results revealed that 73 genes presented increased expression when peak heights were increased (active genes), and 510 genes presented decreased mRNA expression when peak heights were decreased (silent genes, Fig. 4F). These genes were subjected to GO enrichment analysis (Biological Process subitems, BP). The results indicated that active genes are enriched mainly in Postive Regulation of Cell Cylcle, Cell Division, Kinase Activity and Transferase Activity. While silent genes are enriched mainly in subsets such as the ERK1/ERK2 cascade and its regulation, extracellular matrix interactions, and tissue remodelling.

### Runx2 amplifies itself through a positive feedback loop

The ATAC-seq motif prediction results clearly revealed that upregulated Runx2 can directly bind to sequences in gene transcriptional regions, thereby influencing the expression of downstream genes. To elucidate the regulatory relationship between *Runx2* and downstream transcription elements, we performed integrated analysis of Runx2 via ATAC-seq and CUT&Tag of H3K27ac and H3K27me3.

**Fig. 5:**
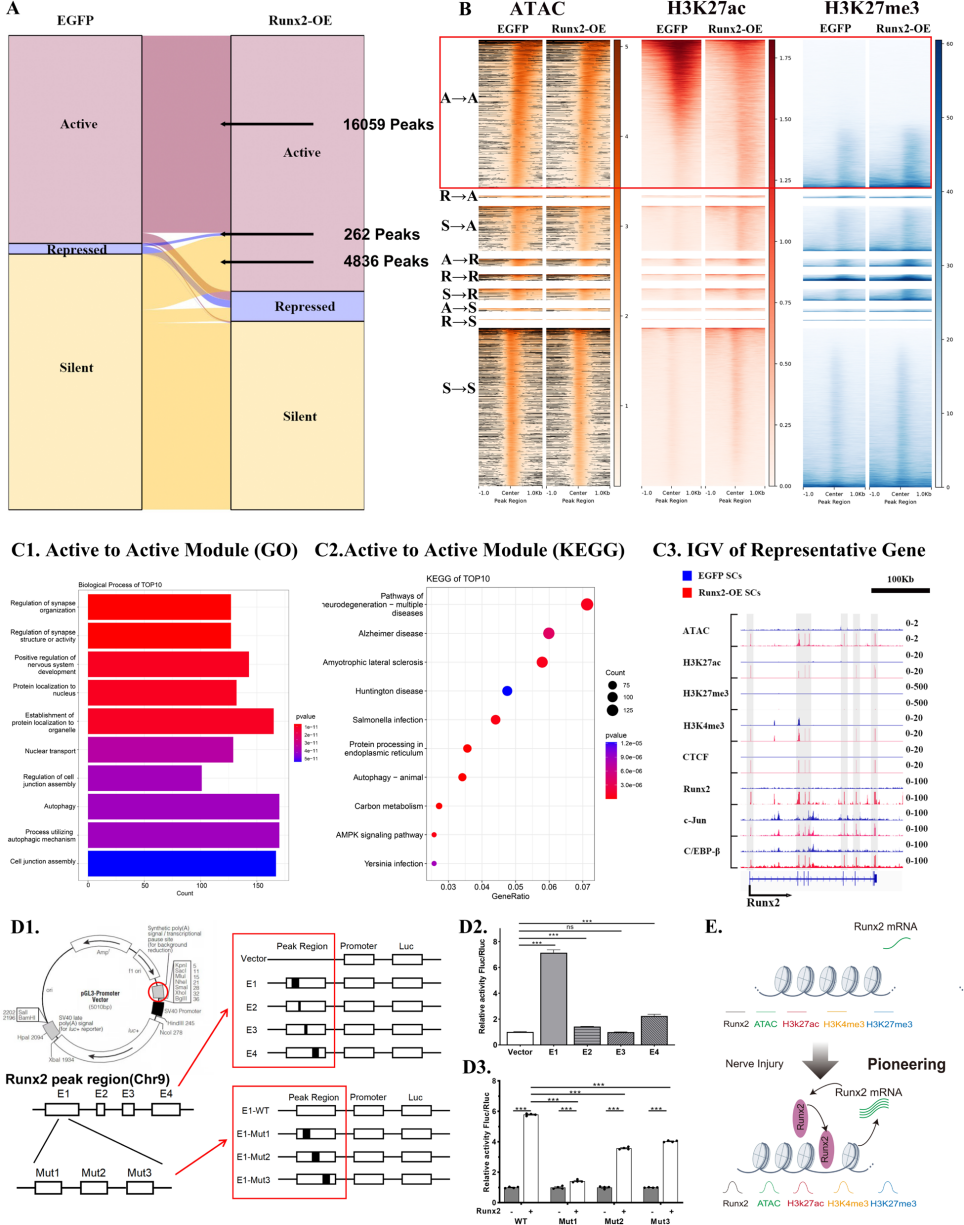
Dynamic Regulation of Transcriptional Activity in Schwann Cells (SCs) Following Runx2 Overexpression (Runx2-OE). Comprehensive analysis of chromatin state transitions based on integrated ATAC-seq and H3K27ac/H3K27me3 Cut&Tag sequencing data.** A** Sankey diagram depicting the transitions in transcriptional activity states proximal to transcription start sites following Runx2-OE.** B** Heatmap visualization depicting the spatial distribution of chromatin accessibility (ATAC) and histone modifications (H3K27ac, H3K27me3) relative to transcription start sites (TSS) across distinct regulatory states after Runx2-OE. Regulatory states are classified as Active (A), Repressed (R), or Silent (S). Red boxes highlight gene sets transitioning from Repressed to Active (RA) or Silent to Active (SA) states. **C** Characterization of genes maintaining Active state (A → A). **C1** Gene Ontology (GO) enrichment analysis. Circle size correlates with the number of differentially expressed genes per category. Color intensity (darker red) indicates statistical significance (p-value). **C2** KEGG pathway enrichment analysis. Circle size represents the number of differentially expressed genes per pathway. Color gradient reflects statistical significance. **C3** Representative genome browser view (IGV) of the Runx2 locus (scale bar = 100 kb). Blue represents the EGFP group, red bands represent the *Runx2*-OE group, and gray areas indicate regions with dramatic peak changes between different tracks. **D** Runx2 autoregulatory mechanism analysis via luciferase reporter assays (n = 4). **D1** Schematic of the luciferase reporter plasmid construction and the *Runx2* peak region sequence; **D2** Bar graph showing the luminescence response in 293 T cells for the Vector control and different elements of *Runx2* peak region (**E1**–**E4**) group; **D3** Bar graph comparing the luminescence response in 293 T cells for mutant versions of the E1 region (Mut1, Mut2 and Mut3) and the wild type (WT) before and after over-expression of *Runx2*; **E** Schematic diagram illustrating the mechanism Runx2 autoregulatory feedback loop. * indicates p < 0.05, ** indicates p < 0.01, *** indicates p < 0.005

On the basis of the comprehensive analysis of all the omics data, the regions regulated after *Runx2*-OE can be classified into three main categories (see Fig. 5A, B):

Transcriptionally active regions (A): ATAC + /H3K27ac + /H3K27me3-

Transcriptionally repressed regions (R): ATAC + /H3K27ac-/H3K27me3 + 

Transcriptionally silent regions (S): ATAC-/H3K27ac-/H3K27me3-

Further analysis of the regions showing changes in transcriptional activity after *Runx2*-OE revealed nine subcategories: A-A, R-A, S-A, A-R, R-R, S-R, A-S, R-S, and S‒S. It could be found that A-A included 16,059 Peaks, R-A included 262 Peaks, and S-A included 4836 peaks (see Fig. 5A). Therefore, we focused mainly on function analysis of these subcategories.

It revealed that *Runx2* itself located in the A-A region (see Fig. 5B and C1–3). Gene Ontology (GO) analysis of the differentially expressed mRNAs (DEmRNAs) in the A-A region revealed enrichment in axons, nervous system development, and autophagy processes (Fig. 5C1). KEGG pathway analysis revealed associations with neurodegenerative processes, autophagy, and the AMPK signalling pathway (Fig. 5C2). HOMER motif prediction indicated a high proportion of Runt family-related transcription factors, especially Runx2 (Fig. 4E3), suggesting that *Runx2* overexpression might be subject to positive feedback regulation, leading to exponential increases in expression levels.

To verify the regulatory mechanism of positive feedback control of *Runx2* expression, we employed a dual-luciferase reporter assay. Different luciferase reporter plasmids were cotransfected with either the *Runx2*-OE plasmid into 293 T cells (Fig. 5D1). Statistical analysis of the luciferase reporter system data via the Shapiro‒Wilk test indicated that the data satisfied the assumption of normality (P > 0.05). And the Brown–Forsythe test suggested that the assumption of homogeneity of variances was met (p < 0.05). The results revealed that the fluorescence intensity in the Vector group was significantly lower than that in E1, E2, E4 groups, especially in the E1 group. These resultes suggested that Runx2 can bind to its transcriptional peak region shown in CUT&Tag results, especially E1 region (Fig. 5D2). Therefore, we selected E1 region for further study. After mutation of predicted Runx2 binding region in E1, it was found that upon *Runx2* overexpression, the E1-mut1, E1-mut2 and E1-mut3 group presented a marked decrease in fluorescence intensity compared to that in the E1-WT group, especially in E1-mut1 group (Fig. 5D3).

### Runx2 acts as a pioneer factor to change plasticity of SCs, via altering both histone modifications in chromatin and transcription factor (TF) binding capacity in SCs

Visualization via IGV software of the gene regulatory regions of *Runx2* further revealed abundant Runx2 peaks in *Runx2*-OE SCs, most of which were accompanied by ATAC-seq peaks, yet changes in H3K27ac and H3K4me3 levels were not prominent (see Fig. 5C3). These findings suggest that the phenotypic changes induced by Runx2 may not rely on histone modifications but rather directly target closed chromatin, influencing the open-closed state of chromatin.

We also conducted CUT&Tag analyses for transcription factors such as c-Jun, C/EBP-β, and CTCF in *Runx2*-OE SCs to assess the impact of Runx2 on the binding of transcription factors in SCs. Visualization via IGV software of the gene regulatory regions of *Runx2* in *Runx2*-OE SCs revealed numerous Runx2 peaks accompanied by peaks for c-Jun, C/EBP-β, and even CTCF (Fig. 5C3). These findings suggest that altering *Runx2* expression can influence the recruitment of other transcription factors, thereby affecting the construction of key transcriptional elements and ultimately impacting cellular characteristics.

**Fig. 6:**
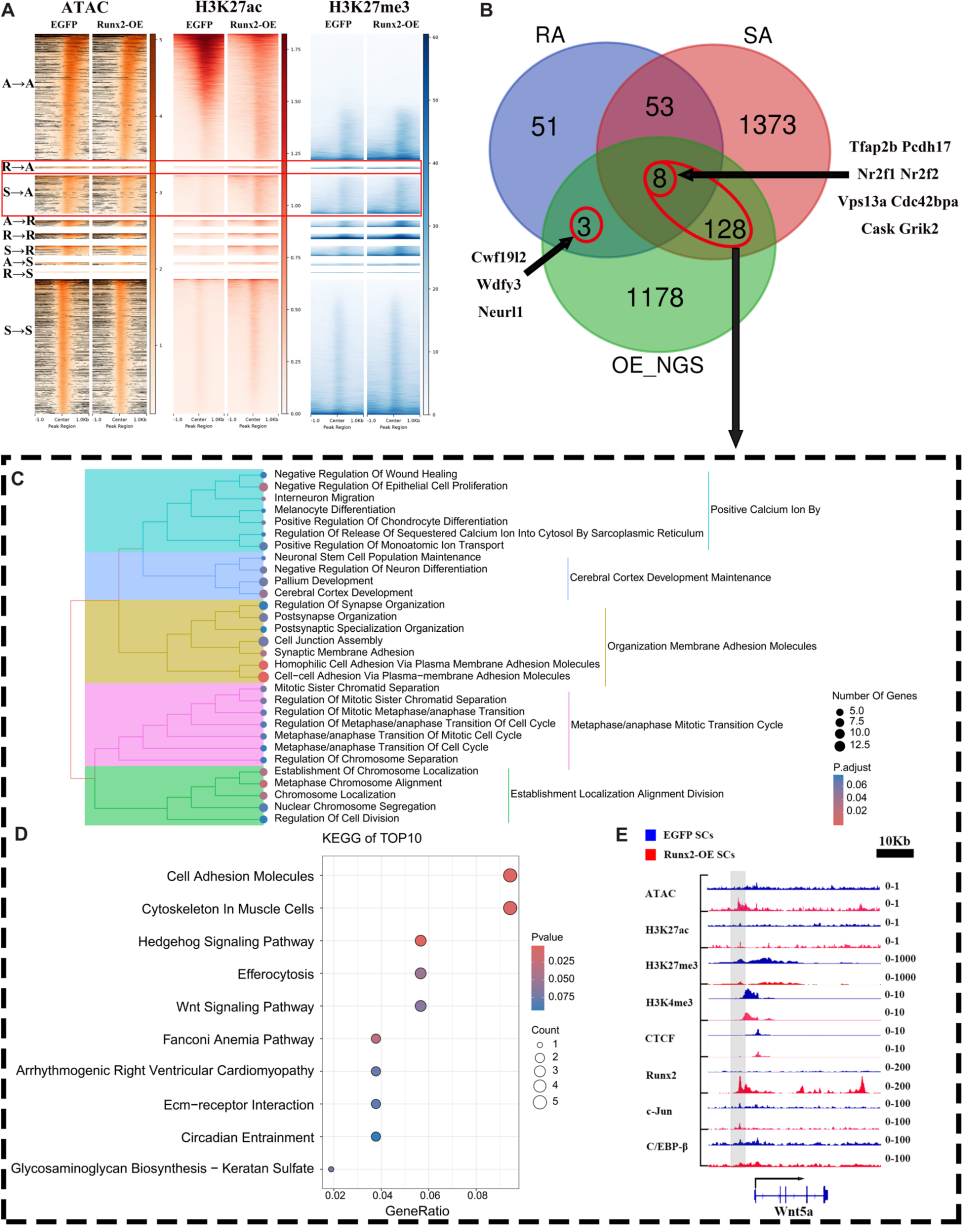
Comprehensive Multi-omics Analysis of Transcriptional Activity Dynamics in Schwann Cells Following Runx2 Overexpression (Runx2-OE). Heatmap visualization depicting the spatial distribution of chromatin accessibility (ATAC) and histone modifications (H3K27ac, H3K27me3) relative to transcription start sites (TSS) across distinct regulatory states after Runx2-OE. Regulatory states are classified as Active (**A**), Repressed (R), or Silent (S). Red boxes highlight gene sets transitioning from Repressed to Active (RA) or Silent to Active (SA) states. Venn diagram illustrating the intersection of three gene sets, including genes transitioning from Repressed to Active (RA), genes transitioning from Silent to Active (SA), upregulated genes following Runx2 overexpression (OE_NGS). Red circle emphasizes the overlapping gene subset. C-E) Functional characterization of genes co-identified in SA and OE_NGS intersections. **C** Gene Ontology (GO) enrichment analysis. Circle size correlates with the number of differentially expressed genes per category. Color intensity (darker red) indicates statistical significance (p-value). **D** KEGG pathway enrichment analysis. Circle size represents the number of differentially expressed genes per pathway. Color gradient reflects statistical significance. **E** Representative genome browser view (IGV) of the Wnt5a locus (scale bar = 10 kb). Blue represents the EGFP group, red bands represent the *Runx2*-OE group, and gray areas indicate regions with dramatic peak changes between different tracks

In order to further verified our assumption, we further focused on genes in R-A and S-A subcategories (see Fig. 6A). When these genesets were combined with up-regulated genes expressed in Runx2-OE SCs (OE_NGS), there were 8 genes showed intersection among these three genesets, including *Tfap2b, Pcdh17, Nr2f1, Nr2f2, Vps13a, Cdc42bpa, Cask and Grik2.* At the same time, we identified three genes (*Cwf1912, Wdfy3, Neurl1*) in the RA and OE-NGS intersection, and 128 genes in the SA and OE-NGS intersection (see Fig. 6B)*.*

Further analysis of 136 genes intersecting SA and OE-NGS revealed that, in Biological Processes, these genes were mainly enriched in the Positivie Calcium Ion, Synapse organization, Adhesion Molecules, Mitotic Cell Cycle, and Cell Division (see Fig. 6C). They mainly executed their function via Hedgehog Signaling Pathway, The Wnt Signaling Pathway, ECM Receptor Interaction, and Cell Adhesion Molecules (see Fig. 6D).

Visualization via IGV software of the gene regulatory regions of its representative gene, *Wnt5a*, further revealed abundant Runx2 peaks in *Runx2*-OE SCs, most of which were accompanied by ATAC-seq and H3K27ac-seq peaks, with decrease changes in H3K27me3 binding (see Fig. 6E). Yet changes in TF levels (c-Jun, C/EBP-β, CTCF) were not prominent.

### Runx2 activates Sox2 and synergistically promotes rapid phenotypic transition of SCs after nerve injury

**Fig. 7:**
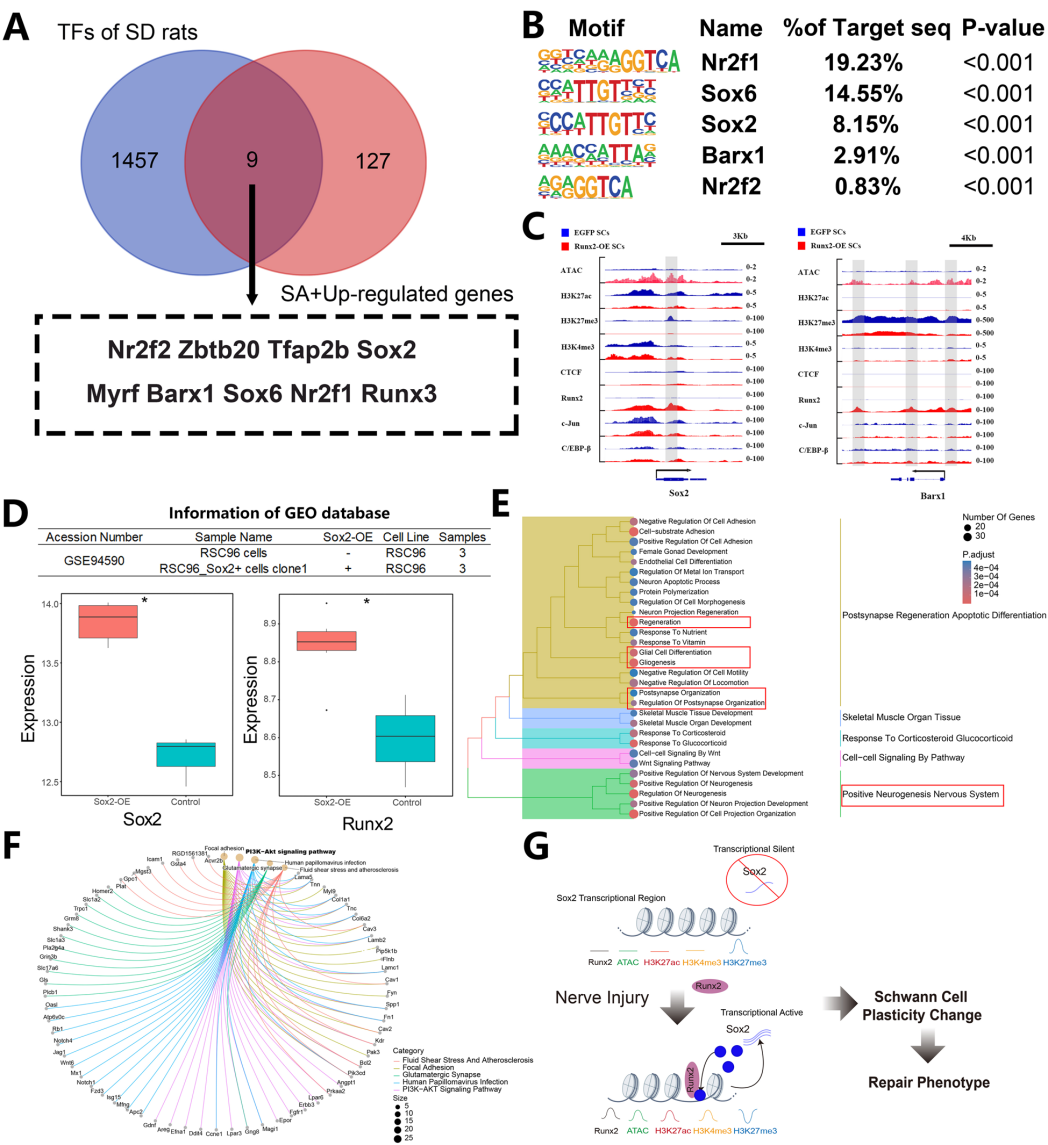
Analysis of Runx2-Associated Key Transcription Factors and Regulatory Mechanisms of Sox2 in Schwann Cells (RSC96). Data source: GSE94590.** A** Venn diagram showing the overlap between upregulated RA genes and common TFs in SD rats. The arrow indicates the list of intersecting genes.** B** HOMER motif prediction from Runx2 CUT&Tag-seq data in Runx2-OE SCs, analyzing the intersecting genes identified in (A).** C** IGV visualization of Sox2 (scale bar = 3 kb) and Barx1 (scale bar = 4 kb). Blue represents the EGFP group, red bands represent the *Runx2*-OE group, and gray areas indicate regions with dramatic peak changes between different tracks.** D** Box plots showing Sox2 and Runx2 expression levels following Sox2 overexpression (Sox2-OE) in RSC96 Schwann cells.** E** GO enrichment analysis (Biological Process) of upregulated genes in Sox2-OE RSC96 cells. Red boxes highlight subcategories of interest. Circle size corresponds to the number of differentially expressed genes in each subcategory. Color intensity (darker red) indicates smaller p-values.** F** KEGG enrichment analysis of upregulated genes in Sox2-OE RSC96 cells. Bold text indicates pathways of interest. Different colored lines represent relationships between pathways and molecules. Circle size corresponds to the number of differentially expressed genes in each pathway.** G** Schematic diagram illustrating the mechanism of Sox2 transcriptional activation by Runx2. * indicates p < 0.05, ** indicates p < 0.01, *** indicates p < 0.005

As searching for TFs in the 136 genes mentioned above, we found 9 TFs that were significantly activated after *Runx2*-OE, including *Nr2f2*, *Zbtb20*, *Tfap2b*, *Sox2*, *Myrf*, *Barx1*, *Sox6*, *Nr2f1*, and *Runx3* (see Fig. 7A). HOMER scanning in the Runx2 Cut&Tag seq results revealed that TFs such as *Sox6*, *Sox2*, *Barx1*, *Nr2f1* and *Nr2f2* can bind to upregulated Peaks after *Runx2*-OE (see Fig. 7B). Visualization via IGV software of the gene regulatory regions of *Sox2* and *Barx1*, It could be observed that an upregulation of Runx2 and ATAC signals accompanied by a decrease in H3K27me3 signal in their transcriptional regulatory region (see Fig. 7C). From these results, we speculated increasing expression of *Runx2* would activate *Sox2* and *Barx1* transcription and increase their expression. As TFs, *Sox2* and *Barx1* would synergistically work with Runx2 by binding to the Runx2 region.

Considering this speculation, we obtained microarray data of RSC96 cells overexpressing *Sox2* (*Sox2*-OE) with the accession number of GSE94590 from GEO database. It was found that in *Sox2*-OE RSC96, the expression of *Sox2* and *Runx2* increased significantly (see Fig. 7D). Biological Process enrichment analysis of upregulated differentially expressed genes in *Sox2*-OE cells revealed that they were mainly enriched in Gilal Cell Differentiation, Giogenesis and Positive Neurogenesis entries (see Fig. 7E). KEGG analysis suggested that the PI3K-AKT pathway might play a role as the main signaling pathway of these actions (see Fig. 7F).

These comprehensive analysis provide insights into the complex regulatory mechanisms influenced by *Runx2* overexpression in SCs, highlighting its role in modulating Schwann Cell plasticity, particularly acting as a pioneer transcription factor. Runx2 executed its function by activating a series of silent/repressed TFs, such as Sox2, synergistically promotes rapid phenotypic transition of SCs after nerve injury (see Fig. 7G).

## Discussion

In the early stages of peripheral nerve injury, Schwann cells (SCs) dedifferentiate from mature SCs to activated repair SCs, which help clear myelin debris (axons and myelin sheaths) produced by Wallerian degeneration, sorting axons and address obstacles in the regeneration pathway. However, the potential inappropriate generation of new myelin sheaths by SCs during this process represents a new obstacle in the regeneration pathway and may prevent axons from going through this pathway. Therefore, precise regulation of the timing of phenotypic transformation of SCs is crucial for nerve regeneration().

### Injury-related SCs phenotype changes and classification

Jessen et al. proposed that following peripheral nerve injury, myelinating SCs are reprogrammed into repair (Bungner) SCs (Gomez-Sanchez et al. 2015). Kalinski et al. performed scRNA-seq on a mouse sciatic nerve injury model and reported that at 3 days post-injury, SCs accounted for 17.48% of the total cell population at the injury site and were categorized into three primary types: SC1, SC2, and SC3(Kalinski et al. 2020). Lovatt et al., through scRNA-seq, identified five subtypes of SCs in rat sciatic nerves following crush injury: Remak SCs, Myelinating SCs, Dividing SCs, Repair SCs, and Transition SCs (Lovatt et al. 2022). These three studies present divergent classifications of postinjury SCs, with varying definitions even for Repair SCs. The Repair SCs initially proposed by Jessen et al. encompass the SC1-3 cell types later described by Kalinski, as well as the Dividing SCs, Repair SCs, and Transition SCs proposed by Lovatt. To facilitate subsequent discussion, a unified classification system for postinjury SCs subtypes is necessary.

In the present study, reprogrammed SCs after injury were referred to as Remak SCs and repair (Bungner) SCs, which were further subdivided into three types: He, HZ, and Zhu SCs (markers shown in STable 4). The transcription regulators enriched in He SCs included Ki67 among others. Functionally, He SCs resemble immature SCs; however, they are a type of repair SCs that typically exhibit high expression levels of *Jun *(Arthur-Farraj et al. 2012) as well as morphology difference. This is in contrast to immature SCs, where *Jun* expression is relatively low. Consequently, this distinction further differentiates He SCs from immature SCs. Zhu SCs are enriched in transcription regulators, including Sox4, Runx2, Hmga1, and Jun, similar to the promyelinating cell state in terms of developmental stage (Kalinski et al. 2020). Repair (Bungner) SCs that were not considered Remak SCs, He SCs, or Zhu SCs were categorized as HZ SCs. In the following text, we use this new classification method to describe the features of each of these cell types.

### Mature SCs can sequentially dedifferentiate into Zhu SCs and He SCs to repair nerve injury

SCs play important roles in nerve axon regeneration. They not only provide scaffolding and neurotrophic factors for nerve regeneration but also guide the orderly extension of axons (Shefa and Jung 2019). During development, SCs evolve from common precursor cells into two morphologically and functionally distinct types: myelinating cells and nonmyelinating cells (Remak SCs). The former cells form myelin around nerve axons (Parmantier et al. 1999).

We obtained scRNA-seq data on rat sciatic nerve crush injury from the GEO dataset GSE216665 (Lovatt et al. 2022). The SCs were annotated using the markers Sox10 + , Plp1 + , and S100b + , and a reclassification of SCs phenotypes was performed. In the early stages of injury, the main categories identified were Mki67^+^ He SCs, Ngfr^+^SOX2^+^ Zhu SCs, and HZ SCs, whereas proportion of myelinating SCs marked by Mbp^+^Mpz^+^Prx^+^ was noticeably reduced. Pseudotime analysis revealed that, in normal nerves, myelinating SCs (yellow) and Remak SCs (purple) are the predominant components. Early post-injury, the of proportion myelinating SCs gradually decreased, and these SCs dedifferentiated towards repair SCs, with Zhu SCs (green) appearing earliest, followed by He SCs (red) (Fig. 7). By day 60, the proportions of He SCs, Zhu SCs, and HZ SCs gradually decreased to levels similar to those of normal nerves, suggesting that in the early stages of injury, SCs sequentially dedifferentiated into repair SCs, including Zhu SCs and He SCs, which participate in the processes of nerve Wallerian degeneration and axonal regeneration. These SCs subtypes exhibit distinct functional roles during the dedifferentiation process. Zhu SCs share similarities with pro-myelinating SCs, maintaining a primed state ready to engage with regenerating axons and initiate remyelination. In contrast, He SCs primarily function to expand the SCs population through proliferation, compensating for SCs loss (through death or apoptosis) during peripheral nerve repair. Notably, He SCs retain the plasticity to revert back to Zhu SCs, demonstrating the dynamic nature of SCs fate transitions during nerve regeneration.

Our studies revealed that *Runx2* expression exhibits temporal dynamics following nerve injury. Current research on the role of Runx2 in the nervous system is focused on its expression in central nervous system gliomas (Yamada et al. 2018) and astrocytes, with relatively few studies on its function in the peripheral nervous system(Li et al. 2021; Hung et al. 2015; Ding et al. 2018; Wang et al. 2022; Hu et al. 2024). Hu et al. created a sciatic nerve crush model in *Runx2* knockout (*Runx2*-KO) mice and reported that the development of the sciatic nerve in *Runx2*-KO mice was not significantly affected. However, in the early stages after nerve injury, the proliferation ability of SCs in KO mice significantly increased, whereas their migration ability decreased (Hu et al. 2024). In vitro, upregulating the expression of *Runx2* in SCs changed the morphology of primary SCs from elongated, spindle-shaped proliferative He SCs to flat, round pro-myelin Zhu SCs, with reduced cell proliferation but no significant increase in apoptosis. We also found that in *Runx2*-OE SCs, the expression of the myelin-related genes *Mbp, Mpz*, and *Pmp22* was significantly downregulated. These findings suggest that elevated Runx2 expression may drive phenotypic switch in SCs.

Previous studies have revealed extensive interactions between *Runx2* and other transcription factors in regulating SCs plasticity (Hung et al. 2015). Hung et al. performed ChIP sequencing analysis on injured peripheral nerves in rats and reported that after activation, c-Jun binds to the enhancer sequences of *Runx2*, inducing the upregulation of *Runx2* expression and participating in the regulation of SCs activation and myelin breakdown and reorganization (Hung et al. 2015). The transcription factors c-Jun, Sox2 and Egr2 are important transcription factors that regulate SCs differentiation states (Jessen and Mirsky 2005). In current study, qPCR revealed that after *Runx2*-OE, the expression of *Jun* and *Egr2,* was significantly downregulated. However, inhibiting *Runx2* had no significant effect on the expression of these genes. Our qRT‒PCR analysis of the injury model revealed that the *Runx2* expression trend was similar to that of *Sox2*, a gene associated with SCs activation/dedifferentiation, but opposite that of *Egr2*, a differentiation-related gene. These interactions form a sophisticated transcriptional network that controls SCs fate determination and phenotypic transitions.

Our studies revealed that at day 3 post-injury (PI3d), *Runx2* expression was first detected in Zhu SCs, corresponding to the earliest appearance of Zhu SCs (Zhu1) in the pseudotime trajectory (green dots above node 2 in Fig. 2 G—PI3d). Subsequently, Runx2 expression in SCs (Zhu2) gradually decreased as He SCs emerged (red dots within the red circle in Fig. 2 G—PI12d). By day 60, Runx2 expression in Zhu SCs had declined to levels comparable to other SCs subtypes. Eventually, all SCs differentiated into either myelinating SCs or Remak SCs. This phenomenon might be attributed to that *Runx2* is closely associated with both the generation and functional maintenance of Zhu SCs.

### Runx2 promotes its own high expression via positive feedback

To gain a deeper understanding of the specific mechanism underlying the epigenetic effects of Runx2, we used CUT&Tag sequencing to assess histone methylation and identify related transcription factors. A comprehensive analysis (Fuglerud et al. 2022) was carried out on ATAC-seq data, H3K27ac, H3K4me3, H3K27me3 data and data for TFs such as Runx2, C-Jun, C/EBP-β, and CTCF; the regions regulated after *Runx2*-OE can be categorized into three main types: transcriptional activation zones (A, ATAC + /H3K27ac + /H3K27me3-, transcription regions remaining active after *Runx2*-OE), transcriptional repression zones (R, ATAC + /H3K27ac-/H3K27me3 + , transcription regions that were repressed after *Runx2*-OE), and transcriptional silencing zones (S, ATAC-/H3K27ac-/H3K27me3-, transcription regions remaining silent regardless of *Runx2*-OE). We found that there was an active region that was obviously dependent on *Runx2* transcription near the coding area of *Runx2*. Visualization of the *Runx2* gene revealed ATAC-seq and H3K27ac peaks in the transcribed region of *Runx2* and peaks corresponding to transcription factors such as c-Jun and C/EBP-β. Moreover, motif prediction of the binding region in *Runx2* revealed that transcription factors related to the Runt family, especially Runx2, occupied a very high proportion of the predicted motifs. These findings indicate that Runx2 can open chromatin and recruit other transcription factors to exert a synergistic effect.

Through dual-luciferase reporter assays, we discovered that Runx2 has the capacity to bind to the transcriptional region of its own gene. This binding initiates a positive feedback loop, resulting in a significant amplification of its own expression levels. These effects contributed to promoting the differentiation and maintenance of Zhu SCs.

### Runx2 is a pioneer transcription factor (pTF) that alters cell plasticity through rousing and cooperating with Sox2

pTFs, also known as pioneer factors, are a class of major transcription factors that can affect chromatin opening and closing, thereby affecting gene expression (Larson et al. 2021). pTFs can recruit different transcription factors at multiple levels and regulate epigenetic changes during the growth and development of various organisms, as well as during the occurrence and development of diseases (Balsalobre and Drouin 2022). Although biological functions involve the synergistic action of many different mechanisms, pTFs, as the main regulatory factors involved in epigenetic regulation, can accurately modulate various mechanisms and change the fate of cells. On the one hand, pTFs can recognize and bind target DNA sequences within closed chromatin, which cannot be bound by most other TFs; on the other hand, after binding to target sequences, pTFs can reshape chromatin, alter the opening and closing of chromatin, promote or inhibit the binding of other TFs and modified proteins to DNA sequences, and trigger biological effects that affect cell fate (Stewart-Morgan et al. 2020). Most targets of pTFs are enhancer sequences located between genes or in introns.

In current study, we found that Runx2 expression was significantly upregulated following nerve injury, predominantly in Schwann cells (SCs) at PI3d and PI12d, correlating with the repair phenotype of Zhu SCs. Through multi-omics analysis, it was established that Runx2 modulated chromatin conformation and histone modification states. On one hand, Runx2 established a positive feedback loop by binding to its own regulatory domains. On the other hand, it promoted the expression of other transcription factors (TFs) and recruits them to regulatory regions for coordinated regulation. Multi-omics analysis revealed nine factors that transitioned from a transcriptionally silent state to an activated state with increased expression following Runx2 overexpression. Combined with HOMER Motif analysis of Runx2 CUT&Tag experiments, we identified five TFs (Nr2f1, Nr2f2, Sox2, Sox6, and Barx1) that potentially exhibit co-binding or adjacent binding relationships with Runx2 PEAKS.

Sox2 is a crucial factor in SCs differentiation regulation. Le et al. demonstrated that Sox2 is primarily expressed in immature SCs, where its overexpression inhibits SCs differentiation, promotes cell proliferation, and suppresses myelination (Le et al. 2005). Parrinello et al. revealed through in vivo and in vitro experiments that Sox2-dependent transcriptional regulation in SCs induces N-cadherin redistribution, stimulating the formation of SCs cords at injury sites and guiding orderly axon regeneration (Klein 2010). Further functional analysis revealed that in Sox2-overexpressing (Sox2-OE) Schwann cells (RSC96), differentially expressed upregulated genes were primarily enriched in pathways related to glial cell differentiation states, neuroregeneration capacity regulation, and PI3K-AKT signaling (Parrinello et al. 2010). Therefore, Runx2 may promote SCs phenotype transition and maintenance of the Zhu SCs phenotype by activating Sox2 expression and co-binding to effector gene regulatory regions.

Moreover, Sox2-OE resulted in increased expression of both Sox2 and Runx2. We hypothesize that elevated Sox2 levels may enhance Runx2 expression, enabling rapid coordinated response of both factors to ensure timely conversion and maintenance of the SCs repair phenotype following nerve injury.

These findings have significant implications for both basic and translational research. From a basic research perspective, our findings enhance our understanding of how Schwann cells facilitate axon regeneration and remyelination through various phenotypic transitions during Wallerian degeneration, thereby enriching the theoretical framework of peripheral nerve regeneration. For translational applications, our findings suggest new approaches to neural tissue engineering through genetic modification, potentially enabling precise control over peripheral nerve regeneration processes. This could lead to optimized regenerative outcomes and eventual clinical applications.

## Conclusion

SCs undergo sequential dedifferentiation into Zhu SCs and He SCs under the influence of Runx2 after injury, predominantly in Zhu SCs. Runx2 can arouse and recruit downstream stemness factors, such as Sox2, by changing the chromatin accessibility and histone modification status within SCs. Therefore, phenotype transformation of SCs could be timely accomplished and accurately maintained after injury.

## Supplementary Information

Supplementary material 1: Figure S1. Construction of the *Runx2*-OE plasmid and *Runx2* sequence information. Table S1. Primer sequences. Table S2. Luciferase reporter sequence of *Runx2 *enhancer. Table S3. Luciferase reporter sequence of Runx2 mutation. Table S4. Specific gene markers of different SCs identified via scRNA-seq. Table S5. List of reagents and antibodies used for experimental detection. Table S6. Important equipment used in the experiments. Software and manufacturer List.

## Data Availability

The data that support the findings of this study are available from the corresponding author upon reasonable request. Transcriptomic and cut&tag sequencing data GSE271351, GSE271353, GSE271356 Series records at: https://www.ncbi.nlm.nih.gov/geo/query/acc.cgi?acc = GSE271351 https://www.ncbi.nlm.nih.gov/geo/query/acc.cgi?acc = GSE271353 https://www.ncbi.nlm.nih.gov/geo/query/acc.cgi?acc = GSE271356.
