# Supplementary material for: Runx2 drives Schwann cells repair phenotype switch through chromatin remodeling and Sox2 activation after nerve injury
Source: Mol Med. 2025 Mar 21;31:110. doi: 10.1186/s10020-025-01142-4 (PMC11929166; doi:10.1186/s10020-025-01142-4)
Supplement: Supplementary file 1 — Supplementary material 1: Figure S1. Construction of the Runx2-OE plasmid and Runx2 sequence information. Table S1. Primer sequences. Table S2. Luciferase reporter sequence of Runx2 enhancer. Table S3. Luciferase reporter sequence of Runx2 mutation. Table S4. Specific gene markers of different SCs identified via scRNA-seq. Table S5. List of reagents and antibodies used for experimental detection. Table S6. Important equipment used in the experiments. Software and manufacturer List. [file 10020_2025_1142_MOESM1_ESM.docx]

**Supplementary information**


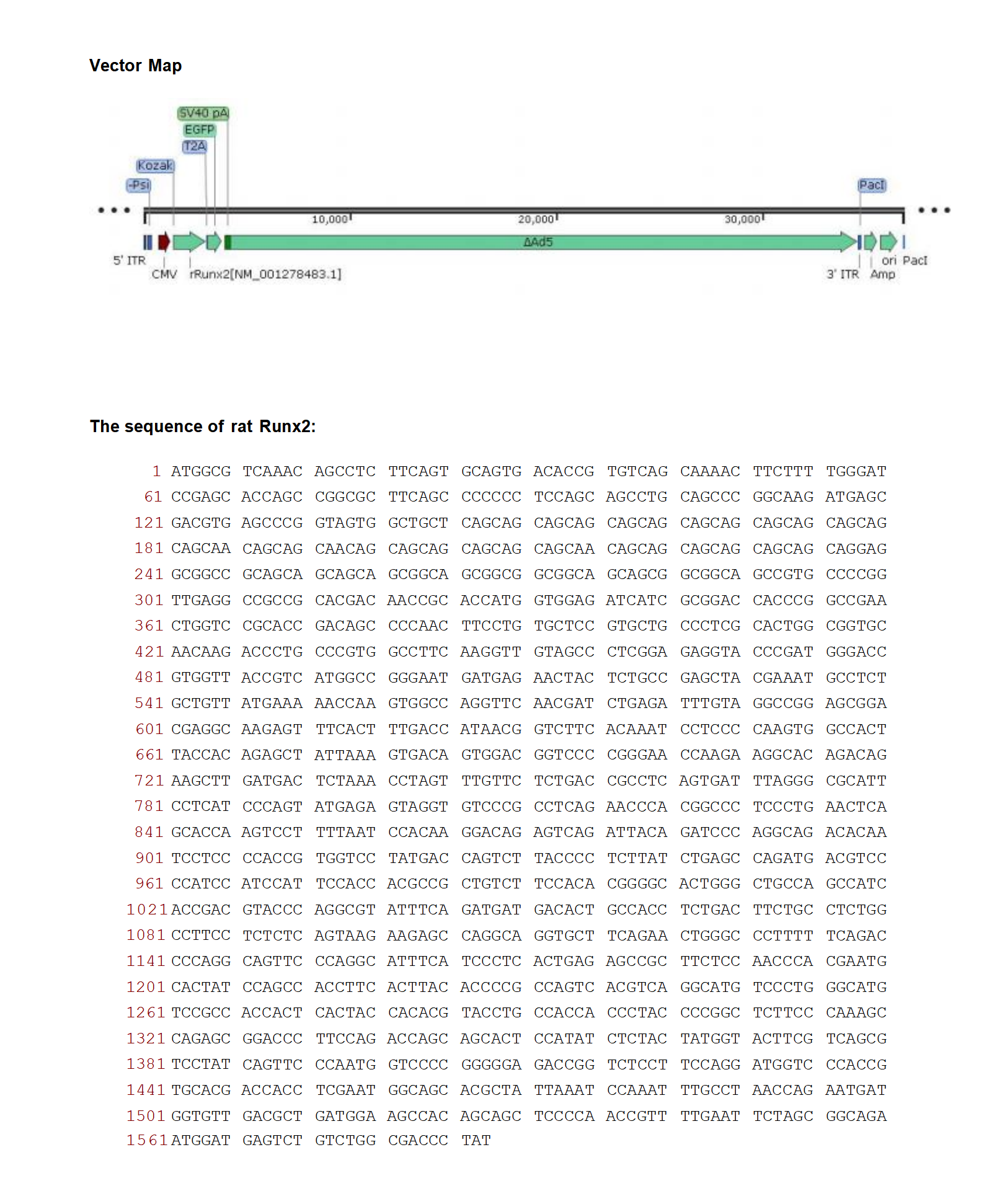


**Figure S1**: Construction of the *Runx2*-OE plasmid and *Runx2* sequence information.

**Table S1.** Primer sequence

| Gene | Forward（5‘-3’） | Reverse（5‘-3’） |
| --- | --- | --- |
| Gaphd | ACA GCA ACA GGG TGG TGG AC | TTT GAG GGT GCA GCG AAC TT |
| Runx2 | CCT AAA TCA CTG AGG CGG TCA G | TGG CCA CTT ACC ACA GAG CTA |
| Egr2 | CCA AGG CCG TAG ACA AAA TCC | TGC CCA TGT AAG TGA AGG TCT G |
| Jun | CTT CTA CGA CGA TGC CCT CAA C | GGG TCG GTG TAG TGG TGA TGT G |
| Sox2 | CAT GAC CAG CTC GCA GAC CTA | CTG GAG TGG GAG GAA GAG GTA A |
| Mbp | CAA GAA CTA CCC ACT ACG G | GTA CGA GGT GTC ACA ATG T |
| Pmp22 | GAT CCT GTC TGT CAT CTT CA | CCA TAG GAG TAG TCG TTG TT |
| Mpz | TTA CAC GGA CAG GGA AGT | CTT GGC ATA GTG GAA GAT TG |

**Table S2.** Luciferase reporter sequence of Runx2 enhancer

| Site | Length (bp) | Sequence |
| --- | --- | --- |
| E1 | 2745 | CAGTGGTCCCAGACTGGACCGGGAGCTCTAGAGACAAGGAGGCCACTCAGCACGAGGAAGTCgtgtgtgtgtgtgtgtgtgtgtgtgtgtgtgtgtgtgtgtgAGTGATAGCTAGTGGCACAGAATTGCTTTCCAGTGGCATAGAGGAGCAGGCAGTGgaggaagagggagactgagaggcgcagggagagtctgagaggaagaTTAGTGGAAGAATCCGGAAAGAGAGCAAACAAGAGACAAGGAAAGACCGACAGAGGGGCATAGTTGTAGGTAGAGCGAGCAGAAAGGGGTGGCCGAAACAGTGACTCAAAGGGGAAGGGCATAGGAGTGCTCCCCAGTGTCATCCTTATGCCTGTGAGAGGGCATGCAGTCACACCCAGCTCTGTCCTCTCCTCAGTCCCCAGAGCGCCTCCCAGGCACAGGAACACAGGTCTCCAGATCATCTGAGCACAAAAATCCACTTTTTGTACTCGCCAAGTTCTTAGCGCACAGAGGCACCAACAACCTCTTTGGCGCCCTGGGGACCTTTTCTATGCTCTgcgggcgcgcgggcaggctgggggggggggggcTCAGAGAGGCAGGATCCTGACAGGTCTCTTGCAGAACTTGGGAGGACTTTGCACCCAACAATAAAAGCACCAATTAGTGGCTATGTGAAGAGTTTTAAATAATGATGGATGGTGAGAAATAAGTTACCTCTTCAGTCCTCTAAGGGGACTACTTCATTGATTTCACCAAATCCCAGTTGTGTCAACTACCTTGTTAGGAGCTACCGGGACTTTGGTAGATGATTCCATTTGGAAGCTTTGGGAGTTTGAAAAGAATCGGTTAAGCAAGACATAACAGACATCACACAAGAGGTGAGCGGGCCCGATATTGCTTCTGCCTAGTTCTTGGGGTGTAGCAGCCTCCGGCGGATTTCCCGGCTTCTGTGGGCGCCGCAAGTTGTTTGATTTGTTTTGAAGGCTCAGAATTTGAGGCTGGTCGGAGACACCCACGTGCTTCTGACTCCCATCAGCATAATGATCGCCTTAGCTTGGGTCGTGTCTATATAAACCACAAAAACCTAATCATTAGAAATCCCAGCCTCCAAAAACCACATTTTAGGTAAAAACTGCTGCTTTTTTCCCCCCACACCCCTTCATCTCTCAACCACAACCTTTTGGGGATTCCAAAATCGCCTACCCCCAAAACGCCGGGaaacaaatacataaataaataattaCTGAACCATTTTTTACCTTTTACTTTTATTCCTCCAAAGTGGGGAGCGGCGAGGAAGACAGTTTAAGACACTAGACTTAGTCTGAGACGAAGACTGGACTTAATAAAAGATTGGAAAATTAATGGATAACCGCTGTGTATTTCCCCTTTCTCAGCAAGCGTTTTTGCAACTTACAACTTATTTTAATTACCAAGCTGTCTTTTTTTTATTTCTTAAAAAAAAAAAAGCAACCCATCCTTATTCTCCAGTAATGACAAGAAGGACTGTATAATAAAAATGTCCAGACATCCGTTAGAGTTTCTAAAACTATTTTGACATGCCCTCCTGTCACCCTTTTCTGATGTCACCTAAGGGGGGAAAAACCACCCCCGAAGTCTGTAAAAACCTTCCCCCCCCCCCCGCCCCAGCCAATgtggggtggggttgggggggaagggtgAAGAGTGCTCAGAAGTCAACTCGGACACTTGAGAATTTTCCTTCCTCCACTCCTGAAGTTATAACGAAAAATTAACGTCAGCAGGAGCAGCCTGAGACTCTCTAGCTTCTCAGCTTCATCGTAGTCAGATCGAGAAGAGGTTCTTGGTCCTGGGGGTGAAGGGGGAGCGGAGGACGCAGGCGGCGATGTCCTAGGCGGGGACCGCCTCCTTCCAACTTCGggcgccgagcgcagcgtgccggcgcttctggcggccggcgggcggcggcagcggctgcgATCCGCAGCTCCAGATCTGTCGCCCCGAGATCCGCTCCCCCCCACCCCCCACTTACCTCCGGGCACCTTGAAACGCCAGGGGGGCCCGGGGCACTTGGCAAAGAGCTGGAGGGACCGGGCTGCGAGGTAGACGGAGGCGAGTGAGAGGGAGAGAGAAACGGGGCAAGGAAGGACTCGGCGGCCGGAGGACTCGGAGCGCGCAGGCAGCGAGGGGAGCGCGCAGCGGCCTCGGAGGAGGAGAAGGAGGCGCCGGCAGGCGACGGCGCCGCGAGCTGGACAGCCACGCTCGGCTTGGCGGTGGCGGACAGCGAAGGGTCACACGCGCCGCCGAGATGGACTGCTGAACCTGCCGGGCTCCACTACGGAACTGGAACCGGGAGTGGGTCGTCAGCGCACCTAGGACACGGTGCCCCGAGGGGCCCCACAACTGCATGCTGCTAACTTCAAGTCCCTTGCCTCGGCTGGGACAAACACGCCCCCGAGTCCCGCTCGCTGCTGCCACCGCCGGGGGCTCCCAAGCCTCCCGGTGGAGATCGGCCCCGCTCGGGTGTCCCCACCCGCCACCCGACTGTACGGggtcgccagccgccggcccccagctgactccgccggcACTCGGCTTTCTCGAACTTGATTTTCTCACCTCTTCTGGCGATCACCTCCATCCTCTTCCCCCTCCCGGCCCGCGTCTCGCCCACCACCTCGATTTCCTCCAcctcgccccccattccacccctcccccgtcccccggccACTTCGCTAACTTTGTGGGCTGTTGTGATGCGTATTCCCGTAG |
| E2 | 650 | TTGTCAGGGAGAGTGGCTAGAGACCTTAACTTTGAAGACAGTCTCTAAGCACTTTCATCATTCTATTCTAGAAATAGTTTTGGCCTAAACCAGATAGTATATGGTTAGGAAAAACGATGAATCAGCAATCTTTCCCAACAGAGACGAGCGACTCATCAATTTTACCGTGGGAAATGCCTCCCAGCTTTTCTCCCCCTCCTTTCTGCCTTCCACTGGACAAAGGGAAAGATAATTTCAAGTTTGTCTTGGTTCTCTGTTAGTTCAATAGGTGCTTGTATGCACGTCCACTAAGATTGTGGTAGATAAAACAGCCTCAAGGGATGGCTAGTCGAGGTGAAGGGGTATCTGTAGAATATAAGGCTTTGAAATTTAGGAATATTCAACGTCAAGTTGCAGCAGACTGACGCTTGCTGAATGCCTCTGGTACCCAAGTATAACCACACGCCATGGACATAACGTGTGGTTGGGCTGTGCTCACTTACCAAGGTAGAAACCAGAGGGAGTCACACAGACAGAAAACACCAGGGTAGCATGAGTCCAGCCTGGGGAGGAGAATTTTGCCAAGTGCAGTTGATGTGCGAATGAATGCATGTTAAGTTCTGGGGACAGCTTGGACAAGGACATCAGACTTCTTTGAAGAGCTGTGGAAG |
| E3 | 950 | agtgagttgaaggtctgtagggtgaatgatggagaccctgtttcaaaaTATAAAGTAGAAGAgggctaggaatagctaactcagtggaagagtgcctgcctagcatgtagaaggctccaggttcaaatcccagtaTGGAGGAAGCGAGGGGGGGTGGAAGGCATGGAGACGGAGACCAAGGCAAATGAAGTGGGGAAGTGTGGTTACTAGAGAAAGTGGAAGGGGTCCAGGTTGACCTGGAGCATACAAGCCACACATGAACAAGAAAGTCATGGGGTCATGGTTTCTAGGATCTAGGTTTGGGGCCTTGGGCAGTTCGGTCTGTACAGCATACTCTAGGGTCTACACTGGCTCATCACCATGCAAAGCGCTCTCAGTCTATCTTGGAGAACCATAGGTTCTAACCACAGGTTAGAGTTTGCATGATTCTGTCTTGGGGTCATCTGCCATCTGGAACCAACTCCTCCTTCCCCTGGCTTGTCTTGTGGATCTGTGGGGTTTGTGAGACgtctcagttgcttttctattgctgtggtgaaacaccatggccaaggcaacttacagaagaaagtttttctttggggcttaagcttcccaagggttgtgttccgtgccgtcagggacagcagatagcagacaggggcagctggagttggaagctgagggctcacatctcaaaccacaaacaggaaacagagccaatagggaatggtatgaggcctcaaagtctgtccccagtgacacatttcctcccaccaggctgtgcctcctaagcctcccagctaagcaccagcaactggggattccaatgcttgagaatgatgggcacatgggggtagggacgaacagggggtatctcattcaaaccatcTCGGAGGGCTAGTCTAAGGCTGGGTTGGGCTTTGATGTGGAGGGGCCACGTTATAGGACGAATGTCATACTCTATCCAA |
| E4 | 2305 | AGTCTGTCAACCGTGCATGGCCCAGTGGCATGCAGTGGCCACGTCCCACATGTGTTAATATATACATATATAAAGAGAGTGCCTATATATGTATATTGATTAGCTATCTAGAAGATTTCTCATTCACTCCCCAGTTGTGATCTGGCAACCCTAAGAGTGTGGGGACAGTCATTACTGGGTTTCATATTGTTTACTATTTAAGATGTCTCCTCTACCAAGGAGCAGACCGTCAAAGGTGTTATCTGGTCTGGTTTCGTAAGTGACCTGTTCCCACAACAATTCAGAGAGGTGGACTCTGGGTCCGGGAGGAAGAACGGCCACTTCCTCCCTGTGCTTTGCAACCAAATCAAGGCCTCTGCTGTGTGGAGGCCTGTGCACTCTGCAGACCAGCTTACAAAGCTGTGGTGCACTCGGAAAGGGACGAGAGGCAGCGTGGCTGCTTCCCCTACCGTCCACTCTGAATACCTGTTCCAAAGCTTCCCTTCAGACTTGATGCAGGTATGCGTTTGAACTTTTGAGTTCACTCtttttttttttttttttCTATTCTAAGAAAGTGACTTCAAAAATACTGATCAGGACAGATTATTTTATTTTACTTTTTTAAAATATTCCCTCACTTCCCCCATTTAACCAAAAAATAAGTCCCATTCCCCTCCCCCGTGCCTCCTGCTTCTCCCTTTATGCAAAACTGAAAATGGCAATATCTTATTATAGCCATAATGATATAGTGTTTGAGTTGGCTGTGTGTTGTTTGTTTTTACTTTCTTTTTTTCCTTTTTTAAGTTATGAATATGTGTAAAATCTGAAGTAACTTGCTAACGTGAATGGTCATATAACTTTAAAGATATATTTATAATTATTTAATGACATTTGGACATTTGGAACATTTCTTAGTGTAATGGTCTGTTGACTTCGGTCTCTAAAAGTGCTTCTTCTTAAATAACAAGTTTCTTCAGTGGGCTAGAGCCATATCGGAAATATTGCTAAGCAATTTCAATTCCTTCAGGCATAATGTGATTTTTTTTTTTTATGATAACTCCCATTTCCAAATATTTTAGTTGTAGTTTTGTTTCCATGATGTATGAAGGAAATGCTATGCTTCTTTCTTTCAGGTGTTTGATTGCCTCTGACACGGCTTTACCTGTTAAAGCAATAATTAGGGATTaaaaaacaaaacaaaacaaaacaaaaaCCACCTATAACCCTGCAGCCCTTTGACGCTTAATGCGGCCTCTTTGCTAGCATTGAAATGCTGGAGACATGTGGTTTCCTAATTTCTTCATTTTTGGGGGGTGGGGGGTTGGCCATTATGAGTCTTGTCATATTAAAAAGCCAAGCACAAGTGATTGGCCGAACTGCAGAAAAGTGTTCTGTGGTCTCAGAGTTGAGCAGAACTCTAAATTGCAGGCTTCGTGGTTGAGGGCCTAGGCAGCTGAAAGCCACACGTGTAGTAAAGGCTCAACCATGACACGTACAGCCTAGGAACACAGATGCATTAGGAGTCTGCACCCCCCCCCCAGCCTTACCACACAGCCATTCAGGGGAACCCAAAAGTGCCTTACCCAAGGAGCCCCCACCCCCAACAGCAGCTTTGCAGGAAGTGGAATGAAGTCAATGTCCTAGGGGAGCTGGTCAGCTGAAGTAGTAGCTGATGTCAGTAGACACAGAGCCTGTGGGGACCTCCAGGAAGCCTTTGATACTCAAGGCTCTCACTAGGGAGGGCCGTAGAGAGCAGGGAAGACAATGTATGTAGCAGTTGGTGTCTGCCATCGTGTGTGAACATAAGATCCAGCCCTCCTAGGAGGACGTACTGTGATCATTCCTGGCACCTTGTGGCCAATCCCTGAGTGTGACTCCCTAATCCAAGCCAGAGATCATTCAGTGACACCACCAGGCAAGTGCTAGCATTCCTCCTGCACAAAGTGTGTTTGTTGGGGGTTGGGGTTGGGGGCCACCTTCCTTTGAATCCAGAGCCATTCTAAGAGTCCTTCAGGCTTTTGCCTTTAGCCCCTACACCCCCTTGCTCTCTGTTCCTTCTCAGGTTGACCTTTGTCCCAATGTGGGACAGTCCAGGGGCAGATGGGGAACTTATGTGTGCCTCCAACCTGTGTTTTCCCTCAGGATTCATCCTGACTCCTTCTGACACAAATTGAGGGGGAAAGAAACCCACCCAGTAGCAAACCGAAACACTTTGCCTTCTAAAGGTTGTGTACCAAGTATGCACAGATAGATGGTCAGGCCACCTTTGTGTTTCTTGAGATGGAATTTGTAGCTGATGCTATTTATTGTCTGTGTGTGGTAG |

**Table S3.** Luciferase reporter sequence of Runx2 mutation

| Site | Length (bp) | Sequence |
| --- | --- | --- |
| E1-WT | 2745 | CAGTGGTCCCAGACTGGACCGGGAGCTCTAGAGACAAGGAGGCCACTCAGCACGAGGAAGTCgtgtgtgtgtgtgtgtgtgtgtgtgtgtgtgtgtgtgtgtgAGTGATAGCTAGTGGCACAGAATTGCTTTCCAGTGGCATAGAGGAGCAGGCAGTGgaggaagagggagactgagaggcgcagggagagtctgagaggaagaTTAGTGGAAGAATCCGGAAAGAGAGCAAACAAGAGACAAGGAAAGACCGACAGAGGGGCATAGTTGTAGGTAGAGCGAGCAGAAAGGGGTGGCCGAAACAGTGACTCAAAGGGGAAGGGCATAGGAGTGCTCCCCAGTGTCATCCTTATGCCTGTGAGAGGGCATGCAGTCACACCCAGCTCTGTCCTCTCCTCAGTCCCCAGAGCGCCTCCCAGGCACAGGAACACAGGTCTCCAGATCATCTGAGCACAAAAATCCACTTTTTGTACTCGCCAAGTTCTTAGCGCACAGAGGCACCAACAACCTCTTTGGCGCCCTGGGGACCTTTTCTATGCTCTgcgggcgcgcgggcaggctgggggggggggggcTCAGAGAGGCAGGATCCTGACAGGTCTCTTGCAGAACTTGGGAGGACTTTGCACCCAACAATAAAAGCACCAATTAGTGGCTATGTGAAGAGTTTTAAATAATGATGGATGGTGAGAAATAAGTTACCTCTTCAGTCCTCTAAGGGGACTACTTCATTGATTTCACCAAATCCCAGTTGTGTCAACTACCTTGTTAGGAGCTACCGGGACTTTGGTAGATGATTCCATTTGGAAGCTTTGGGAGTTTGAAAAGAATCGGTTAAGCAAGACATAACAGACATCACACAAGAGGTGAGCGGGCCCGATATTGCTTCTGCCTAGTTCTTGGGGTGTAGCAGCCTCCGGCGGATTTCCCGGCTTCTGTGGGCGCCGCAAGTTGTTTGATTTGTTTTGAAGGCTCAGAATTTGAGGCTGGTCGGAGACACCCACGTGCTTCTGACTCCCATCAGCATAATGATCGCCTTAGCTTGGGTCGTGTCTATATAAACCACAAAAACCTAATCATTAGAAATCCCAGCCTCCAAAAACCACATTTTAGGTAAAAACTGCTGCTTTTTTCCCCCCACACCCCTTCATCTCTCAACCACAACCTTTTGGGGATTCCAAAATCGCCTACCCCCAAAACGCCGGGaaacaaatacataaataaataattaCTGAACCATTTTTTACCTTTTACTTTTATTCCTCCAAAGTGGGGAGCGGCGAGGAAGACAGTTTAAGACACTAGACTTAGTCTGAGACGAAGACTGGACTTAATAAAAGATTGGAAAATTAATGGATAACCGCTGTGTATTTCCCCTTTCTCAGCAAGCGTTTTTGCAACTTACAACTTATTTTAATTACCAAGCTGTCTTTTTTTTATTTCTTAAAAAAAAAAAAGCAACCCATCCTTATTCTCCAGTAATGACAAGAAGGACTGTATAATAAAAATGTCCAGACATCCGTTAGAGTTTCTAAAACTATTTTGACATGCCCTCCTGTCACCCTTTTCTGATGTCACCTAAGGGGGGAAAAACCACCCCCGAAGTCTGTAAAAACCTTCCCCCCCCCCCCGCCCCAGCCAATgtggggtggggttgggggggaagggtgAAGAGTGCTCAGAAGTCAACTCGGACACTTGAGAATTTTCCTTCCTCCACTCCTGAAGTTATAACGAAAAATTAACGTCAGCAGGAGCAGCCTGAGACTCTCTAGCTTCTCAGCTTCATCGTAGTCAGATCGAGAAGAGGTTCTTGGTCCTGGGGGTGAAGGGGGAGCGGAGGACGCAGGCGGCGATGTCCTAGGCGGGGACCGCCTCCTTCCAACTTCGggcgccgagcgcagcgtgccggcgcttctggcggccggcgggcggcggcagcggctgcgATCCGCAGCTCCAGATCTGTCGCCCCGAGATCCGCTCCCCCCCACCCCCCACTTACCTCCGGGCACCTTGAAACGCCAGGGGGGCCCGGGGCACTTGGCAAAGAGCTGGAGGGACCGGGCTGCGAGGTAGACGGAGGCGAGTGAGAGGGAGAGAGAAACGGGGCAAGGAAGGACTCGGCGGCCGGAGGACTCGGAGCGCGCAGGCAGCGAGGGGAGCGCGCAGCGGCCTCGGAGGAGGAGAAGGAGGCGCCGGCAGGCGACGGCGCCGCGAGCTGGACAGCCACGCTCGGCTTGGCGGTGGCGGACAGCGAAGGGTCACACGCGCCGCCGAGATGGACTGCTGAACCTGCCGGGCTCCACTACGGAACTGGAACCGGGAGTGGGTCGTCAGCGCACCTAGGACACGGTGCCCCGAGGGGCCCCACAACTGCATGCTGCTAACTTCAAGTCCCTTGCCTCGGCTGGGACAAACACGCCCCCGAGTCCCGCTCGCTGCTGCCACCGCCGGGGGCTCCCAAGCCTCCCGGTGGAGATCGGCCCCGCTCGGGTGTCCCCACCCGCCACCCGACTGTACGGggtcgccagccgccggcccccagctgactccgccggcACTCGGCTTTCTCGAACTTGATTTTCTCACCTCTTCTGGCGATCACCTCCATCCTCTTCCCCCTCCCGGCCCGCGTCTCGCCCACCACCTCGATTTCCTCCAcctcgccccccattccacccctcccccgtcccccggccACTTCGCTAACTTTGTGGGCTGTTGTGATGCGTATTCCCGTAG |
| E1-Mut1 | 2745 | CAGTGGTCCCAGACTGGACCGGGAGCTCTAGAGACAAGGAGGCCACTCAGCACGAGGAAGTCgtgtgtgtgtgtgtgtgtgtgtgtgtgtgtgtgtgtgtgtgAGTGATAGCTAGTGGCACAGAATTGCTTTCCAGTGGCATAGAGGAGCAGGCAGTGgaggaagagggagactgagaggcgcagggagagtctgagaggaagaTTAGTGGAAGAATCCGGAAAGAGAGCAAACAAGAGACAAGGAAAGACCGACAGAGGGGCATAGTTGTAGGTAGAGCGAGCAGAAAGGGGTGGCCGAAACAGTGACTCAAAGGGGAAGGGCATAGGAGTGCTCCCCAGTGTCATCCTTATGCCTGTGAGAGGGCATGCAGTCACACCCAGCTCTGTCCTCTCCTCAGTCCCCAGAGCGCCTCCCAGGCACAGGAACACAGGTCTCCAGATCATCTGAGCACAAAAATCCACTTTTTGTACTCGCCAAGTTCTTAGCGCACAGAGGCACCAACAACCTCTTTGGCGCCCTGGGGACCTTTTCTATGCTCTgcgggcgcgcgggcaggctgggggggggggggcTCAGAGAGGCAGGATCCTGACAGGTCTCTTGCAGAACTTGGGAGGACTTTGCACCCAACAATAAAAGCACCAATTAGTGGCTATGTGAAGAGTTTTAAATAATGATGGATGGTGAGAAATAAGTTACCTCTTCAGTCCTCTAAGGGGACTACTTCATTGATTTCACCAAATCCCAGTTGTGTCAACTACCTTGTTAGGAGCTACCGGGACTTTGGTAGATGATTCCATTTGGAAGCTTTGGGAGTTTGAAAAGAATCGGTTAAGCAAGACATAACAGACATCACACAAGAGGTGAGCGGGCCCGATATTGCTTCTGCCTAGTTCTTGGGGTGTAGCAGCCTCCGGCGGATTTCCCGGCTTCTGTGGGCGCCGCAAGTTGTTTGATTTGTTTTGAAGGCTCAGAATTTGAGGCTGGTCGGAGACACCCACGTGCTTCTGACTCCCATCAGCATAATGATCGCCTTAGCTTGGGTCGTGTCTATATGGGTTGTGGAAACCTAATCATTAGAAATCCCAGCCTCCAAAAACCACATTTTAGGTAAAAACTGCTGCTTTTTTCCCCCCACACCCCTTCATCTCTCAACCACAACCTTTTGGGGATTCCAAAATCGCCTACCCCCAAAACGCCGGGaaacaaatacataaataaataattaCTGAACCATTTTTTACCTTTTACTTTTATTCCTCCAAAGTGGGGAGCGGCGAGGAAGACAGTTTAAGACACTAGACTTAGTCTGAGACGAAGACTGGACTTAATAAAAGATTGGAAAATTAATGGATAACCGCTGTGTATTTCCCCTTTCTCAGCAAGCGTTTTTGCAACTTACAACTTATTTTAATTACCAAGCTGTCTTTTTTTTATTTCTTAAAAAAAAAAAAGCAACCCATCCTTATTCTCCAGTAATGACAAGAAGGACTGTATAATAAAAATGTCCAGACATCCGTTAGAGTTTCTAAAACTATTTTGACATGCCCTCCTGTCACCCTTTTCTGATGTCACCTAAGGGGGGAAAAACCACCCCCGAAGTCTGTAAAAACCTTCCCCCCCCCCCCGCCCCAGCCAATgtggggtggggttgggggggaagggtgAAGAGTGCTCAGAAGTCAACTCGGACACTTGAGAATTTTCCTTCCTCCACTCCTGAAGTTATAACGAAAAATTAACGTCAGCAGGAGCAGCCTGAGACTCTCTAGCTTCTCAGCTTCATCGTAGTCAGATCGAGAAGAGGTTCTTGGTCCTGGGGGTGAAGGGGGAGCGGAGGACGCAGGCGGCGATGTCCTAGGCGGGGACCGCCTCCTTCCAACTTCGggcgccgagcgcagcgtgccggcgcttctggcggccggcgggcggcggcagcggctgcgATCCGCAGCTCCAGATCTGTCGCCCCGAGATCCGCTCCCCCCCACCCCCCACTTACCTCCGGGCACCTTGAAACGCCAGGGGGGCCCGGGGCACTTGGCAAAGAGCTGGAGGGACCGGGCTGCGAGGTAGACGGAGGCGAGTGAGAGGGAGAGAGAAACGGGGCAAGGAAGGACTCGGCGGCCGGAGGACTCGGAGCGCGCAGGCAGCGAGGGGAGCGCGCAGCGGCCTCGGAGGAGGAGAAGGAGGCGCCGGCAGGCGACGGCGCCGCGAGCTGGACAGCCACGCTCGGCTTGGCGGTGGCGGACAGCGAAGGGTCACACGCGCCGCCGAGATGGACTGCTGAACCTGCCGGGCTCCACTACGGAACTGGAACCGGGAGTGGGTCGTCAGCGCACCTAGGACACGGTGCCCCGAGGGGCCCCACAACTGCATGCTGCTAACTTCAAGTCCCTTGCCTCGGCTGGGACAAACACGCCCCCGAGTCCCGCTCGCTGCTGCCACCGCCGGGGGCTCCCAAGCCTCCCGGTGGAGATCGGCCCCGCTCGGGTGTCCCCACCCGCCACCCGACTGTACGGggtcgccagccgccggcccccagctgactccgccggcACTCGGCTTTCTCGAACTTGATTTTCTCACCTCTTCTGGCGATCACCTCCATCCTCTTCCCCCTCCCGGCCCGCGTCTCGCCCACCACCTCGATTTCCTCCAcctcgccccccattccacccctcccccgtcccccggccACTTCGCTAACTTTGTGGGCTGTTGTGATGCGTATTCCCGTAG |
| E1-Mut2 | 2745 | CAGTGGTCCCAGACTGGACCGGGAGCTCTAGAGACAAGGAGGCCACTCAGCACGAGGAAGTCgtgtgtgtgtgtgtgtgtgtgtgtgtgtgtgtgtgtgtgtgAGTGATAGCTAGTGGCACAGAATTGCTTTCCAGTGGCATAGAGGAGCAGGCAGTGgaggaagagggagactgagaggcgcagggagagtctgagaggaagaTTAGTGGAAGAATCCGGAAAGAGAGCAAACAAGAGACAAGGAAAGACCGACAGAGGGGCATAGTTGTAGGTAGAGCGAGCAGAAAGGGGTGGCCGAAACAGTGACTCAAAGGGGAAGGGCATAGGAGTGCTCCCCAGTGTCATCCTTATGCCTGTGAGAGGGCATGCAGTCACACCCAGCTCTGTCCTCTCCTCAGTCCCCAGAGCGCCTCCCAGGCACAGGAACACAGGTCTCCAGATCATCTGAGCACAAAAATCCACTTTTTGTACTCGCCAAGTTCTTAGCGCACAGAGGCACCAACAACCTCTTTGGCGCCCTGGGGACCTTTTCTATGCTCTgcgggcgcgcgggcaggctgggggggggggggcTCAGAGAGGCAGGATCCTGACAGGTCTCTTGCAGAACTTGGGAGGACTTTGCACCCAACAATAAAAGCACCAATTAGTGGCTATGTGAAGAGTTTTAAATAATGATGGATGGTGAGAAATAAGTTACCTCTTCAGTCCTCTAAGGGGACTACTTCATTGATTTCACCAAATCCCAGTTGTGTCAACTACCTTGTTAGGAGCTACCGGGACTTTGGTAGATGATTCCATTTGGAAGCTTTGGGAGTTTGAAAAGAATCGGTTAAGCAAGACATAACAGACATCACACAAGAGGTGAGCGGGCCCGATATTGCTTCTGCCTAGTTCTTGGGGTGTAGCAGCCTCCGGCGGATTTCCCGGCTTCTGTGGGCGCCGCAAGTTGTTTGATTTGTTTTGAAGGCTCAGAATTTGAGGCTGGTCGGAGACACCCACGTGCTTCTGACTCCCATCAGCATAATGATCGCCTTAGCTTGGGTCGTGTCTATATAAACCACAAAAACCTAATCATTAGAAATCCCAGCCTCCAAGGGTTGTGCTTTAGGTAAAAACTGCTGCTTTTTTCCCCCCACACCCCTTCATCTCTCAACCACAACCTTTTGGGGATTCCAAAATCGCCTACCCCCAAAACGCCGGGaaacaaatacataaataaataattaCTGAACCATTTTTTACCTTTTACTTTTATTCCTCCAAAGTGGGGAGCGGCGAGGAAGACAGTTTAAGACACTAGACTTAGTCTGAGACGAAGACTGGACTTAATAAAAGATTGGAAAATTAATGGATAACCGCTGTGTATTTCCCCTTTCTCAGCAAGCGTTTTTGCAACTTACAACTTATTTTAATTACCAAGCTGTCTTTTTTTTATTTCTTAAAAAAAAAAAAGCAACCCATCCTTATTCTCCAGTAATGACAAGAAGGACTGTATAATAAAAATGTCCAGACATCCGTTAGAGTTTCTAAAACTATTTTGACATGCCCTCCTGTCACCCTTTTCTGATGTCACCTAAGGGGGGAAAAACCACCCCCGAAGTCTGTAAAAACCTTCCCCCCCCCCCCGCCCCAGCCAATgtggggtggggttgggggggaagggtgAAGAGTGCTCAGAAGTCAACTCGGACACTTGAGAATTTTCCTTCCTCCACTCCTGAAGTTATAACGAAAAATTAACGTCAGCAGGAGCAGCCTGAGACTCTCTAGCTTCTCAGCTTCATCGTAGTCAGATCGAGAAGAGGTTCTTGGTCCTGGGGGTGAAGGGGGAGCGGAGGACGCAGGCGGCGATGTCCTAGGCGGGGACCGCCTCCTTCCAACTTCGggcgccgagcgcagcgtgccggcgcttctggcggccggcgggcggcggcagcggctgcgATCCGCAGCTCCAGATCTGTCGCCCCGAGATCCGCTCCCCCCCACCCCCCACTTACCTCCGGGCACCTTGAAACGCCAGGGGGGCCCGGGGCACTTGGCAAAGAGCTGGAGGGACCGGGCTGCGAGGTAGACGGAGGCGAGTGAGAGGGAGAGAGAAACGGGGCAAGGAAGGACTCGGCGGCCGGAGGACTCGGAGCGCGCAGGCAGCGAGGGGAGCGCGCAGCGGCCTCGGAGGAGGAGAAGGAGGCGCCGGCAGGCGACGGCGCCGCGAGCTGGACAGCCACGCTCGGCTTGGCGGTGGCGGACAGCGAAGGGTCACACGCGCCGCCGAGATGGACTGCTGAACCTGCCGGGCTCCACTACGGAACTGGAACCGGGAGTGGGTCGTCAGCGCACCTAGGACACGGTGCCCCGAGGGGCCCCACAACTGCATGCTGCTAACTTCAAGTCCCTTGCCTCGGCTGGGACAAACACGCCCCCGAGTCCCGCTCGCTGCTGCCACCGCCGGGGGCTCCCAAGCCTCCCGGTGGAGATCGGCCCCGCTCGGGTGTCCCCACCCGCCACCCGACTGTACGGggtcgccagccgccggcccccagctgactccgccggcACTCGGCTTTCTCGAACTTGATTTTCTCACCTCTTCTGGCGATCACCTCCATCCTCTTCCCCCTCCCGGCCCGCGTCTCGCCCACCACCTCGATTTCCTCCAcctcgccccccattccacccctcccccgtcccccggccACTTCGCTAACTTTGTGGGCTGTTGTGATGCGTATTCCCGTAG |
| E1-Mut | 2745 | CAGTGGTCCCAGACTGGACCGGGAGCTCTAGAGACAAGGAGGCCACTCAGCACGAGGAAGTCgtgtgtgtgtgtgtgtgtgtgtgtgtgtgtgtgtgtgtgtgAGTGATAGCTAGTGGCACAGAATTGCTTTCCAGTGGCATAGAGGAGCAGGCAGTGgaggaagagggagactgagaggcgcagggagagtctgagaggaagaTTAGTGGAAGAATCCGGAAAGAGAGCAAACAAGAGACAAGGAAAGACCGACAGAGGGGCATAGTTGTAGGTAGAGCGAGCAGAAAGGGGTGGCCGAAACAGTGACTCAAAGGGGAAGGGCATAGGAGTGCTCCCCAGTGTCATCCTTATGCCTGTGAGAGGGCATGCAGTCACACCCAGCTCTGTCCTCTCCTCAGTCCCCAGAGCGCCTCCCAGGCACAGGAACACAGGTCTCCAGATCATCTGAGCACAAAAATCCACTTTTTGTACTCGCCAAGTTCTTAGCGCACAGAGGCACCAACAACCTCTTTGGCGCCCTGGGGACCTTTTCTATGCTCTgcgggcgcgcgggcaggctgggggggggggggcTCAGAGAGGCAGGATCCTGACAGGTCTCTTGCAGAACTTGGGAGGACTTTGCACCCAACAATAAAAGCACCAATTAGTGGCTATGTGAAGAGTTTTAAATAATGATGGATGGTGAGAAATAAGTTACCTCTTCAGTCCTCTAAGGGGACTACTTCATTGATTTCACCAAATCCCAGTTGTGTCAACTACCTTGTTAGGAGCTACCGGGACTTTGGTAGATGATTCCATTTGGAAGCTTTGGGAGTTTGAAAAGAATCGGTTAAGCAAGACATAACAGACATCACACAAGAGGTGAGCGGGCCCGATATTGCTTCTGCCTAGTTCTTGGGGTGTAGCAGCCTCCGGCGGATTTCCCGGCTTCTGTGGGCGCCGCAAGTTGTTTGATTTGTTTTGAAGGCTCAGAATTTGAGGCTGGTCGGAGACACCCACGTGCTTCTGACTCCCATCAGCATAATGATCGCCTTAGCTTGGGTCGTGTCTATATAAACCACAAAAACCTAATCATTAGAAATCCCAGCCTCCAAAAACCACATTTTAGGTAAAAACTGCTGCTTTTTTCCCCCCACACCCCTTCATCTCTTGGTTGTGGCCTTTTGGGGATTCCAAAATCGCCTACCCCCAAAACGCCGGGaaacaaatacataaataaataattaCTGAACCATTTTTTACCTTTTACTTTTATTCCTCCAAAGTGGGGAGCGGCGAGGAAGACAGTTTAAGACACTAGACTTAGTCTGAGACGAAGACTGGACTTAATAAAAGATTGGAAAATTAATGGATAACCGCTGTGTATTTCCCCTTTCTCAGCAAGCGTTTTTGCAACTTACAACTTATTTTAATTACCAAGCTGTCTTTTTTTTATTTCTTAAAAAAAAAAAAGCAACCCATCCTTATTCTCCAGTAATGACAAGAAGGACTGTATAATAAAAATGTCCAGACATCCGTTAGAGTTTCTAAAACTATTTTGACATGCCCTCCTGTCACCCTTTTCTGATGTCACCTAAGGGGGGAAAAACCACCCCCGAAGTCTGTAAAAACCTTCCCCCCCCCCCCGCCCCAGCCAATgtggggtggggttgggggggaagggtgAAGAGTGCTCAGAAGTCAACTCGGACACTTGAGAATTTTCCTTCCTCCACTCCTGAAGTTATAACGAAAAATTAACGTCAGCAGGAGCAGCCTGAGACTCTCTAGCTTCTCAGCTTCATCGTAGTCAGATCGAGAAGAGGTTCTTGGTCCTGGGGGTGAAGGGGGAGCGGAGGACGCAGGCGGCGATGTCCTAGGCGGGGACCGCCTCCTTCCAACTTCGggcgccgagcgcagcgtgccggcgcttctggcggccggcgggcggcggcagcggctgcgATCCGCAGCTCCAGATCTGTCGCCCCGAGATCCGCTCCCCCCCACCCCCCACTTACCTCCGGGCACCTTGAAACGCCAGGGGGGCCCGGGGCACTTGGCAAAGAGCTGGAGGGACCGGGCTGCGAGGTAGACGGAGGCGAGTGAGAGGGAGAGAGAAACGGGGCAAGGAAGGACTCGGCGGCCGGAGGACTCGGAGCGCGCAGGCAGCGAGGGGAGCGCGCAGCGGCCTCGGAGGAGGAGAAGGAGGCGCCGGCAGGCGACGGCGCCGCGAGCTGGACAGCCACGCTCGGCTTGGCGGTGGCGGACAGCGAAGGGTCACACGCGCCGCCGAGATGGACTGCTGAACCTGCCGGGCTCCACTACGGAACTGGAACCGGGAGTGGGTCGTCAGCGCACCTAGGACACGGTGCCCCGAGGGGCCCCACAACTGCATGCTGCTAACTTCAAGTCCCTTGCCTCGGCTGGGACAAACACGCCCCCGAGTCCCGCTCGCTGCTGCCACCGCCGGGGGCTCCCAAGCCTCCCGGTGGAGATCGGCCCCGCTCGGGTGTCCCCACCCGCCACCCGACTGTACGGggtcgccagccgccggcccccagctgactccgccggcACTCGGCTTTCTCGAACTTGATTTTCTCACCTCTTCTGGCGATCACCTCCATCCTCTTCCCCCTCCCGGCCCGCGTCTCGCCCACCACCTCGATTTCCTCCAcctcgccccccattccacccctcccccgtcccccggccACTTCGCTAACTTTGTGGGCTGTTGTGATGCGTATTCCCGTAG |

**Table S4.** Specific gene markers of different SCs in scRNA-seq.

| Sub-cluster (original article) | Repair | Dividing | Remak | Myelinating | Transition |
| --- | --- | --- | --- | --- | --- |
| Nomination (current article) | Zhu | He | Remak | Myelinating | HZ |
| Marker | Ngfr | Top2a | Egr2 | Mbp |  |
|  | Bdnf | Cdk1 | Gfap | Mpz |  |
|  | Gdnf | Mki67 | Cdh19 | Mag |  |
|  | Erbb3 |  | Scn7a | Prx |  |
|  | Sox2 |  |  | Cldn19 |  |
|  |  |  |  | Egr2 |  |

**Table S5.** List of Reagents and Antibodies Used for Experimental Detection.

| Reagent Name | Manufacturer | Dilution ratio |
| --- | --- | --- |
| Paraformaldehyde (PFA) | McLean | 4:100 |
| Pentobarbital sodium | China Pharmaceutical Shanghai Chemical Reagents Company | 1:100 |
| Goat serum for blocking | Zhongshan Jinqiao, China | 1:10 |
| Hoechst 33342 | H21492, Invitrogen, USA | 1:500 |
| Rabbit anti-rat NF polyclonal antibody | Sigma | 1:200 |
| Alexa 488-labelled goat anti-rabbit IgG antibody | Life Technology | 1:600 |
| monoclonal mouse anti-CD31 antibody | Sigma, Japan | 1:400 |
| monoclonal mouse anti-NF200 antibody | Sigma, Japan | 1:400 |
| Goat anti-Rabbit IgG (H+L) Cross-Adsorbed Secondary Antibody Alexa Fluor® 568 | ThermoFisher, USA | 1:300 |
| Alexa Fluor® 488-conjugated AffiniPure Goat Anti-mouse IgG (H+L) | Jackson ImmunoResearch, USA | 1:400 |
| DAPI | Merck, Germany | 2μg/mL |
| Rabbit anti-rat S-100 polyclonal antibody | Boster | 1:100 |
| Alexa 555-labelled goat anti-rabbit IgG antibody | Life Technology | 1:600 |
| Alexa 647-labelled goat anti-rabbit IgG antibody | Life Technology | 1:600 |
| TUNEL staining kit | Beijing Solarbio | —— |
| EdU Imaging Kits | Invitrogen |  |
| Runx2 siRNA | Ribobio, China | 3'-GGT TCA ACG ATC TGA GAT T-5’ |
| Opti-Men | Gibico, USA |  |
| Lipofectamine3000 Reagent | Thermo, Massachusetts, USA |  |
| Runx2-OE / EGFP vector | Cyagen Biosciences, China |  |
| SMART-Seq ® HT Kit | Clontech |  |
| Hieff NGS® ATAC-Seq Library Prep Kit | Yeasen, China |  |
| Anti-Histone H3 (acetyl K27) antibody - ChIP grade | Abcam, USA | 1:50 |
| Anti-Histone H3 (trimethyl K27) antibody- ChIP grade | Abcam, USA | 1:50 |
| Anti-RUNX2 antibody - ChIP grade | Abcam, USA | 1:50 |
| Recombinant anti-CTCF antibody - ChIP grade | Abcam, USA | 1:50 |
| Rabbit Anti-Mouse IgG H&L: | Abcam, USA | 1:100 |
| RevertAid First Strand cDNA Synthesis Kit | Thermo Fisher Scientific |  |
| FastStart Universal SYBR Green Master Mix | Roche, Switzerland |  |

**Table S6.** Important Equipment Used in the Experiments. Software and Manufacturer List.

| Device/software name (version) | | Manufacturer | |  |
| --- | --- | --- | --- | --- |
| Freezing microtome | | 6 L FREEZONE PLUS, Labconco, USA | |  |
| Stereo microscope | | Carl Zeiss, Germany | |  |
| Laser scanning confocal microscope | | ZESS LSM800,Germany | |  |
| Gene Ontology (GO) | | http://www.geneontology.org | |  |
| Kyoto Encyclopedia of Genes and Genomes Pathway (KEGG) | | http://www.genome.jp/kegg/ | |  |
| Protein Data Bank (PDB) | | https://www.wwpdb.org/ | |  |
| STRING | | https://cn.string-db.org/cgi/input.pl | |  |
| Photoshop CS6 | | Adobe Systems | |  |
| ImageJ | | National Institutes of Health, USA | |  |
| SPSS 20.0 | | IBM, USA | |  |
| GraphPad Prism 9.3 | | GraphPad Software | |  |
| StepOnePlus Real-Time PCR instrument | | Thermo Fisher Scientific | |  |
| Electric constant temperature water bath pot (DK-8D) | | Shanghai Yiheng Technology Co., Ltd | |  |
| Electrophoresis instrument (EPS 300) | | Shanghai Tianneng Technology Co., Ltd. | |  |
| PCR amplifier 2700 | | Applied Biosystems | |  |
| Electronic balance (BS-2000S) | | Beijing Sartorius Instrument Systems Co., Ltd. | |  |
| Constant temperature shaker MTD-8222 | | Jinghong Co., Ltd. | |  |
| Desktop constant temperature shaker SHK-99-Ⅱ | | North Tongzheng Biotechnology | |  |
| Gel imaging system Tanon-1200 | | Shanghai Tianneng Technology Co., Ltd. | |  |
| Microplate reader Molecular Devices SpectraMax M5 | | MD, USA | |  |
| Qubit and Agilent Bioanalyzer 2100 | Agilent Technologies, USA | |  | |
| Bioptic Qsep400 Analyzer | Bioptic Inc., China | |  | |
